# Zinc- and Copper-Loaded Nanosponges from Cellulose Nanofibers Hydrogels: New Heterogeneous Catalysts for the Synthesis of Aromatic Acetals

**DOI:** 10.3390/gels8010054

**Published:** 2022-01-12

**Authors:** Laura Riva, Angelo Davide Lotito, Carlo Punta, Alessandro Sacchetti

**Affiliations:** Department of Chemistry, Materials, and Chemical Engineering “G. Natta”, Politecnico di Milano, 20131 Milan, Italy; laura2.riva@polimi.it (L.R.); angelodavide.lotito@mail.polimi.it (A.D.L.); carlo.punta@polimi.it (C.P.)

**Keywords:** nanocellulose hydrogels, cellulose-based nanosponges, heterogeneous catalysis, acetalization, metal-catalyzed reactions, sustainability

## Abstract

Herein we report the synthesis of cellulose-based metal-loaded nano-sponges and their application as heterogeneous catalysts in organic synthesis. First, the combination in water solution of TEMPO-oxidized cellulose nanofibers (TOCNF) with branched polyethyleneimine (bPEI) and citric acid (CA), and the thermal treatment of the resulting hydrogel, leads to the synthesis of an eco-safe micro- and nano-porous cellulose nano-sponge (CNS). Subsequently, by exploiting the metal chelation characteristics of CNS, already extensively investigated in the field of environmental decontamination, this material is successfully loaded with Cu (II) or Zn (II) metal ions. Efficiency and homogeneity of metal-loading is confirmed by scanning electron microscopy (SEM) analysis with an energy dispersive X-ray spectroscopy (EDS) detector and by inductively coupled plasma-optical emission spectrometry (ICP-OES) analysis. The resulting materials perform superbly as heterogeneous catalysts for promoting the reaction between aromatic aldehydes and alcohols in the synthesis of aromatic acetals, which play a fundamental role as intermediates in organic synthesis. Optimized conditions allow one to obtain conversions higher than 90% and almost complete selectivity toward acetal products, minimizing, and in some cases eliminating, the formation of carboxylic acid by-products. ICP-OES analysis of the reaction medium allows one to exclude any possible metal-ion release, confirming that catalysis undergoes under heterogeneous conditions. The new metal-loaded CNS can be re-used and recycled five times without losing their catalytic activity.

## 1. Introduction

The demand for sustainable and environmentally friendly materials is constantly increasing nowadays. The design and development of products and processes by promoting the reduction or possibly even the abatement of the use of dangerous substances, following the guidelines of green chemistry principles [1], encounters the request of the Sustainable Development Goals [2].

Considering these recommendations, in the last years we have developed a new class of nanocellulose-based nanostructured materials which could be easily produced from aqueous dispersions of TEMPO (2,2,6,6-tetramethylpiperidine-1-oxyl)-oxidised cellulose nanofibers (TOCNF) [3], branched polyethyleneimine (bPEI), and citric acid (CA), by thermally treating the resulting hydrogels according to a two-step protocol, namely freeze-drying, followed by heating at about 100 °C [4]. These cellulose-based nanosponges have shown a high micro-porosity (due to the freeze-drying step), but also a nano-porosity derived from the cross-linking between primary amines of bPEI and the carboxylic groups pf TOCNF [5].

Recently, the synthetic protocol has been revised and optimized following an eco-design approach that is an eco-toxicological evaluation of the material in order to guarantee more and more the eco-safety of our final cellulose nanosponges (CNS) [6]. This is due to the fact that these engineered nanomaterials have been primary developed for being in direct contact with the environment, which are particularly efficient in the decontamination of water solutions from organic dyes and heavy metals [4,6,7]. In particular, the latter can be efficiently trapped and coordinated in the CNS network thanks to the chelating action of the several amino-groups present in the nanostructure and deriving from bPEI cross-linker.

More recently the same material has been successfully proposed as an ideal heterogeneous catalyst for promoting Henry and Knoevenagel reactions due to its alkaline properties [8]. In this context, by considering the high affinity of CNS for heavy metals, as demonstrated when exploiting their decontamination action, we envisioned that the same structure could be considered as a biomass-derived support for metal ions, to be used for promoting metal-catalyzed organic reactions under heterogeneous conditions.

The acetalization reaction is one of the most useful protecting methods for carbonyl compounds in multi-step reactions [9]. Moreover, acetals are important compounds with various practical applications, widely used as flavors and fragrances in the cosmetic and food industry [10]. Despite their simple molecular structure, the practical synthesis of acetals is not trivial, since the equilibrium of the reaction is shifted toward the reagents and the yields are often low.

Most methods for acetal synthesis are based on the reaction of a carbonyl compound with alcohols or ortoesters [11] and these reactions are generally catalyzed using *p*-toluenesulfonic acid, pyridinium salts, triflic acid [12,13,14] and Lewis acids in their metal chlorides and trifluoromethanesulfonates form, as well as transition metal complexes [15,16]. These homogeneous catalysts show several limitations, which include the problematic work-up procedures, the use of toxic and corrosive reagents, the necessity of neutralization of the strong acid media, and the lack of possibility of recovering and recycling the catalyst [17]. This is of particular importance when contamination of the final products is an issue, as in the case of pharmaceutical substances.

A practical way to prevent these issues is the use of heterogeneous catalysis, in which the metal is supported and immobilized on a solid material, thus allowing for the recovery of the catalyst by simple filtration at the end of the process. For these reactions, different heterogeneous catalysts have been already reported in literature, namely clays [18] and resins [19,20,21]. However, most of these heterogeneous systems must be used in a high catalyst to substrate ratio and for long reaction times, which favours the formation of side products. At the same time, the use of carbon-based nanostructures as heterogeneous supports for the catalysis of organic reactions has been attracting more and more attention [22,23].

Therefore, we considered the possibility of loading CNS with two different transition metal cations (Zn (II) and Cu (II)), and to use the new metal-organic composites as heterogeneous catalysts for the acetalization reaction.

## 2. Results and Discussion

### 2.1. Catalyst Synthesis and Characterization

**Scheme 1:**
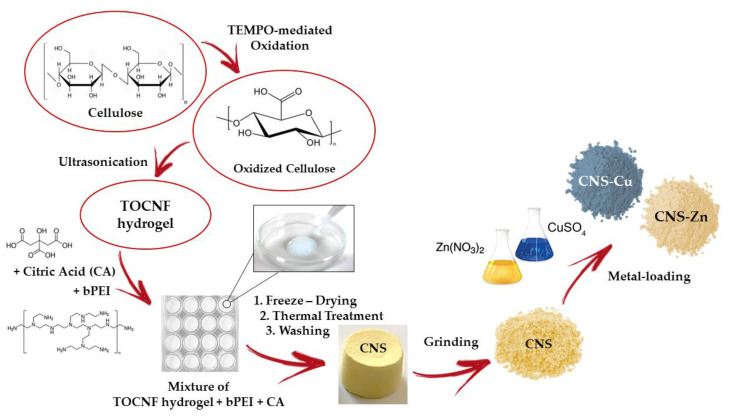
Different production steps for the production of CNS.

The catalyst was synthesized following a previously reported procedure, according to [6,7]. The schematic synthetic procedure is described in Scheme 1.

Briefly, TOCNF and bPEI were first mixed in deionized water in a 1:2 weight ratio, and CA (18% mol with respect to primary amino groups of bPEI) was added to better fix bPEI in the final network by increasing the number of carboxylic groups. The resulting nanocellulose-based hydrogel was transferred in molds, lyophilized, and then heated in oven at about 100 °C, in order to favour the cross-linking by removing water and promoting the formation of amide bonds between the carboxylic groups of TOCNF and CA and the primary amines of bPEI. Finally, CNS were grinded in a mortar before use, in order to increase the exposed superficial area.

CNS so obtained had been previously characterized by several techniques. First, the formation of amide bonds after thermal treatment had been evidenced by FT-IR analysis [24,25] and also confirmed by ^13^C CP-MAS solid-state NMR [4]. Moreover, a high micro-porosity due to the freeze-drying step, with pore sizes in the range of 10–100 μm, had been observed by scanning electron microscopy (SEM) [4], and estimated to be around 70–75% by microcomputed tomography (μ-CT) analysis [24], with trabecular inner structure with an average trabecular thickness of about 30–40 μm and a trabecular separation of about 70–75 μm. Furthermore, nano-porosity in the network had been also revealed by means of small angle neutron scattering (SANS) analysis of water nano-confinement geometries in the sorbent material [5].

Once synthesized, CNS were loaded with Zn (II) and Cu (II) ions, in order to produce the metal-organic composite to be used as heterogeneous catalyst. The loading efficiency was determined by means of ICP-OES and Table 1 shows the percentage in weight of the metals present on the catalyst.

In order to verify the stability of loaded CNS under reaction conditions, we simulated a standard synthetic procedure in a 15 mL vial in absence of reagents and we conducted an ICP-OES analysis on the reaction mixture. The results reported in Table 2 revealed that the amounts of metal released into solution were negligible.

**Figure 1:**
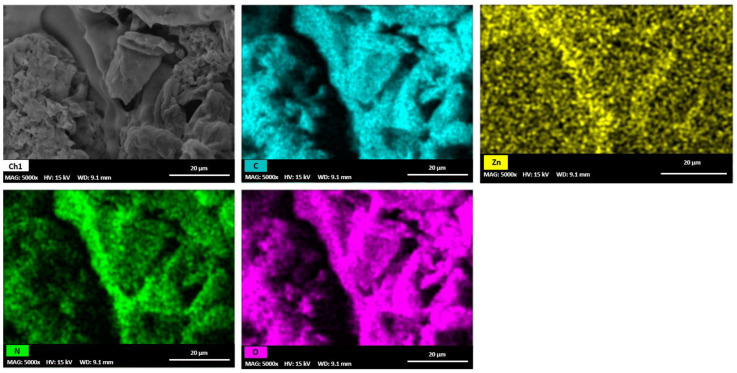
Distribution of elements on CNS-Zn observed with EDS analysis.

**Figure 2:**
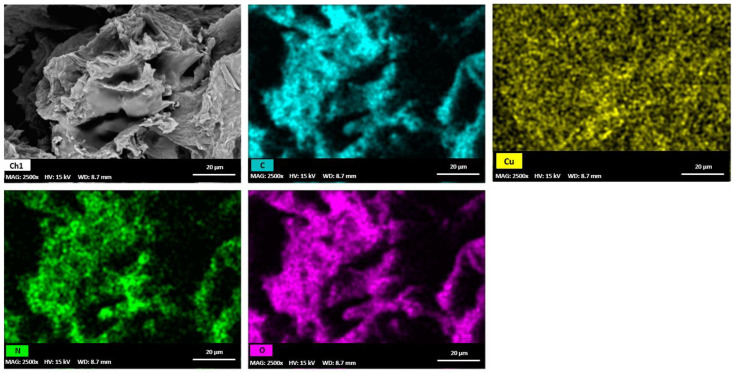
Distribution of elements on CNS-Cu observed with EDS analysis.

The loading efficacy and homogeneity was also confirmed by SEM-EDS analysis (Figure 1 and Figure 2).

### 2.2. Optimization of the Reaction Conditions in Methanol

Initial experiments, devoted to the verification of activity of CNS-Cu and CNS-Zn as heterogeneous catalysts and to the optimization of the protocol, were conducted under different operating conditions by reacting *p*-F-benzaldehyde with methanol under microwave irradiation instead of conventional heating. As expected, the formation of the carboxylic acid product due to the auto-oxidation of the aldehyde in the presence of oxygen could be observed, since the reaction is not conducted in an inert environment. The percentage of carboxylic acid formed is reported in the tables to demonstrate how effective the catalyst is in converting the aldehyde to acetal before its oxidation to carboxylic acid.

In a first set of reactions, the effect of temperature was evaluated (Table 3). Two blank tests in the presence of not-loaded CNS were carried out at 40 °C (*entry 1* of Table 3) and 80 °C (*entry 4* of Table 3), showing that in the absence of metal no formation of any desired product could be observed.

Despite the lower conversion values in the case of the Cu-catalyzed reactions, we decided to work at 40 °C when using methanol as a solvent. This choice was taken since working at 40 °C provides advantages in terms of costs and safety. Moreover, by optimizing the amount of catalyst to be used in order to obtain the desired results, we envisioned it could be still possible to reach high conversions also for CNS-Cu. For these reasons, once the temperature was fixed, we progressively reduced the amount of catalyst (Table 4).

The reactions were repeated starting with 35% *w*/*w* catalyst and gradually reducing the quantity of catalyst up to a 2% *w*/*w* amount.

The results with **CNS-Cu** showed that, by operating at 40 °C, it was possible to achieve very good conversions (*entry 3* in Table 4) using only 2% *w*/*w* catalyst. Unexpectedly, increasing the amount of CNS-Cu catalyst at T = 40 °C resulted in a lower conversion, whereas at T = 80 °C, high conversion was obtained. We can suggest that at lower temperature, an interaction between the aldehyde and the copper loaded nanosponge is established, thus making the substrate less reactive or available and slowing the reaction. This hypothesis could explain why a lower amount of catalyst is beneficial to the reaction. A possible implication of the backbone CNS material was excluded, since with the use Zinc this effect was not observed. Nonetheless, we decided to not further investigate this very unusual outcome, having reached optimal results with the 2% of catalyst loading.

On the other hand, in the case of CNS-Zn it was necessary to work with 15% *w*/*w* catalyst to obtain interesting results. Despite the higher percentage of catalyst, in this case the results were satisfactory also in view of the possibility of recycling it, as it will be discussed later. From the tests conducted, we can therefore conclude that the ideal operating conditions for reactions in methanol were 40 °C and 2% *w*/*w* of catalyst for CNS-Cu, and 40 °C and 15% *w*/*w* of catalyst for Zn-CNS. In fact, under these conditions, both the catalysts led to a selectivity >95% towards product **1** (acetal) with very high conversions.

### 2.3. Kinetic Tests

The model reaction was also followed along the time, with both CNS-Cu (Table 5) and CNS-Zn (Table 6), in order to analyze the course of the reaction in terms of conversion.

**Figure 3:**
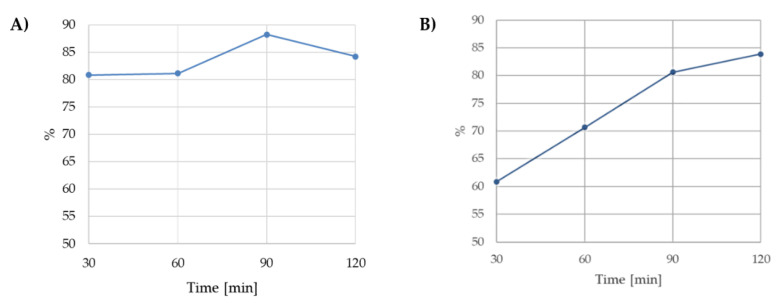
(**A**) Conversion trend for reaction with CNS-Cu; (**B**) conversion trend for the reaction with CNS-Zn.

For reactions with CNS-Cu a good conversion could be observed already after 30 min, while for reactions with CNS-Zn it was necessary to extend the time to 120 min to achieve the maximum conversion (Figure 3). Therefore, in order to standardize the reaction time for both metals, 120 min of reaction time was selected as the optimal condition.

### 2.4. Scope of the Reaction

**Scheme 2:**
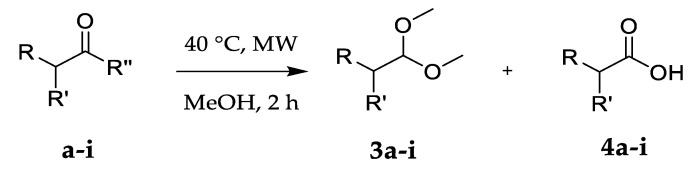
General reaction scheme for the formation of the acetal and the acid product in MeOH.

With the optimized conditions in hand, we set out to examine the scope of the reaction. First, we extended the transformation to a wider range of carbonyl compounds (Table 7). General reaction is reported in Scheme 2.

For the reactions catalyzed by CNS-Cu, selectivity towards product **3** was always almost complete, while conversions were generally quite high, except for some substrates (*entries 6*, *8*, and *10* in (Table 7), but in any case, lower than those obtained in the presence of CNS-Zn. Nevertheless, results obtained with CNS-Cu result interesting if we consider that we worked with a very low percentage of catalyst (2% *w*/*w*). Reactions catalyzed by CNS-Zn showed a high selectivity towards product c too. The conversion in this case turned out to be on average higher than the reactions with CNS-Cu.

Hence, for the choice of catalyst in the case of reactions in methanol it is necessary to decide between higher conversions, thus using CNS-Zn, with the disadvantage of the quantity of catalyst to be used, or to accept slightly lower conversions, but operating with lower quantities of catalyst CNS-Cu.

### 2.5. Optimization of the Reaction Conditions in Ethanol

Finally, we verified if the best conditions developed for methanol could be transferred to ethanol operating with CNS-Cu as catalyst.

In this case we saw a significantly lower conversion in ethanol (Table 8) and consequently we proceeded with the optimization of the reaction by maintaining fixed the percentage amount of the catalyst used (CNS-Cu 2% *w*/*w*) and varying reaction time and temperature (Table 9).

The results suggested that by increasing the temperature up to 80 or 100 °C, the selectivity in all cases was about 80% in favour of product **5**. Therefore, in order to choose the best conditions, we focused on only on the results related to conversion.

**Scheme 3:**
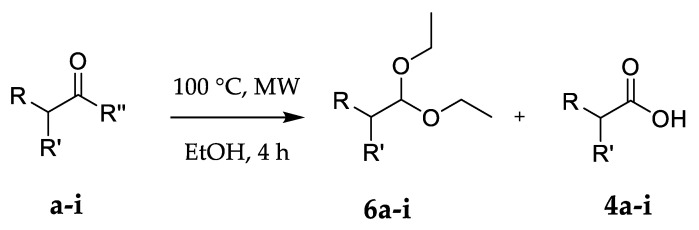
General reaction scheme for the formation of the acetal and the acid product in EtOH.

The operating conditions were selected to be 4 h, 100 °C and 2% *w*/*w* catalyst for CNS-Cu. For CNS-Zn, regarding the percentage of catalyst used, we selected the best conditions found in MeOH (15% *w*/*w*) and choosing the reaction time and temperature conditions that gave the best performance for CNS-Cu, namely 100 °C for 4 h. Scheme 3 reports the general reaction in ethanol.

Once optimized the operating conditions, the reaction was tested on a wide family of carbonyl compounds (Table 10).

The reactions catalyzed by CNS-Cu showed in all cases excellent selectivity, but in most cases the conversion was too low to be considered interesting. The reactions catalyzed by CNS-Zn in addition to excellent selectivity provided high conversions, over 80%, being therefore of greater interest.

Hence, we can conclude that using ethanol as solvent under these operating conditions, unlike in methanol, there is a strong difference in the results obtained with CNS-Cu and CNS-Zn, as only the latter guarantees both high selectivity and high conversion.

### 2.6. Catalyst Recycling and Reuse

**Figure 4:**
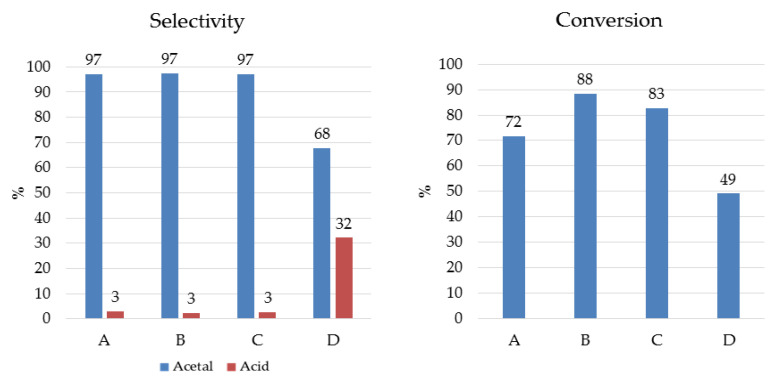
Schematic results for the reusability test with CNS-Cu.

**Figure 5:**
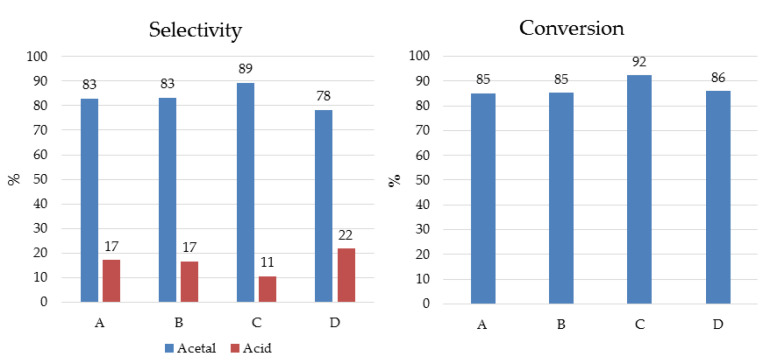
Schematic results for the reusability test with CNS-Zn.

Referring to the optimized conditions in methanol, we also conducted some tests to verify the possibility to recover and re-use the CNS-metal catalyst at the end of the reaction, by filtering the mixture, drying the solid at about 50 °C for 2 h and then re-using it. Four reusability cycles were performed on both CNS-Cu and CNS-Zn metal-loaded catalysts and results are reported in Table 11 and Table 12, respectively, while Figure 4 and Figure 5 show schematically the reusability test results for the same materials.

Results confirmed that both the catalysts could be reused without any loss of performance in terms of selectivity and conversion, at least for three cycles. In the case of CNS-Cu at the fourth recycling test, there was a decrease in both selectivity and conversion. However, this result could be ascribed to the difficulty in recovering the entire amount of catalyst from the third test, by considering the significantly lower quantities used in this case (2% *w*/*w* versus 15% *w*/*w* of CNS-Zn).

## 3. Conclusions

In this work, we investigated the possibility of converting the use of CNS aerogels, obtained by combining TOCNF with bPEI and CA in a unique hydrogel, from a material for water decontamination from heavy metal ions to a totally green heterogeneous support for metal-catalyzed organic reactions. Exploiting the metal chelation characteristics of this aerogel, previously highlighted in the field of environmental remediation, it was possible to load the catalyst with Cu (II) and Zn (II) metal ions and to test the performances of the resulting composites in heterogeneous catalysis for the synthesis of aromatic acetals. The loading efficiency and homogeneity was verified by both ICP-OES and SEM-EDS analysis.

Using methanol as a solvent, we were able to obtain high conversions and good selectivity towards acetal products under optimized conditions, which comprised of operation in a microwave reactor, at low reaction times and temperatures never higher than 100 °C, and with low percentages of the catalysts which, in the case of CNS-Cu, reached a minimum value of 2%.

The scope of the reaction, first tested on *p*-F-benzaldehyde as a starting substrate, was extended to a wider range of aromatic carbonylic compounds, obtaining (in all cases) high yields in the desired acetal products, with in just a few cases tiny amounts of carboxylic acids as by-products. In order to further investigate the general interest of this reaction, ethanol was also used as solvent and reagent. Also in this case, after a required optimization of the reaction conditions, good results were observed both in terms of conversion and selectivity.

The catalysts could be recovered and re-used up to five times without a significant loss in the catalytic activity. In fact, no leaching of metal ions in the reaction medium was observed after ICP-OES analysis of the organic solution.

Due to the high conversions and selectivity observed, the recyclability of the material, and its production starting from sustainable and bio-based renewable sources, the proposed heterogeneous system candidates are much more convenient with respect to both homogeneous and heterogeneous catalysts proposed in the literature for the synthesis of aromatic acetals.

## 4. Materials and Methods

All of the reagents were purchased from Merck (Darmstadt, Germany). Cotton linter was obtained from Bartoli paper factory (Capannori, Lucca, Italy). Deionized water was produced within the laboratories with a Millipore Elix^®^ Deionizer with Progard^®^ S2 ion exchange resins (Merck KGaA, Darmstadt, Germany). All ^1^H-NMR spectra were recorded on a 400 MHz Brüker (Billerica, MA, USA) NMR spectrometer. Microwave reactions were conducted in a Biotage^®^ Initiator+ (Uppsala, Sweden). Other equipment used in the procedures include a Branson SFX250 Sonicator (Emerson Electric Co., Ferguson, MI, USA), a SP Scientific BenchTop Pro Lyophilizer (SP INDUSTRIES, 935 Mearns Road, Warminster, UK), a Büchi Rotavapor^®^ R-124 8 (Flawil, Switzerland) and a Thermotest—Mazzali laboratory oven (Monza, Italy). Scanning electron microscopy (SEM) was performed using a variable-pressure instrument (SEM Cambridge Stereoscan 360) at 100/120 pA with a detector BSD. The operating voltage was 15 kV with an electron beam current intensity of 100 pA. The focal distance was 9 mm. The EDS analysis was performed using a Bruker Quantax 200 6/30 instrument (Billerica, MA, USA). The metal concentrations were measured by ICP-OES atomic emission spectroscopy using a Perkin Elmer Optima 3000 SD spectrometer (Wellesley, MA, USA).

### 4.1. TEMPO-Oxidized Cellulose Nanofibers (TOCNFs) Production and Titration

100 g of cotton linter paper were minced with gradual addition of deionized water. Simultaneously, 2.15 g of tetramethyl-piperidine-*N*-oxide (TEMPO) and 15.42 g of KBr were dissolved in 2 L of deionized water. Once the paper was homogeneously blended with water, the solution was transferred in the reaction keg and water was added in order to obtain a total volume of 5.7 L. Then, 437 mL of 12% *w*/*v* NaClO aqueous solution were dripped in the reaction mixture, keeping the pH above 10.5–11.0 with dropwise addition of NaOH 4 M. The suspension was left stirring overnight and after 12–16 h the oxidized cellulose was acidified with HCl 12 N until a pH of 1–2 to induce the aggregation of the cellulose fibers and their easy separation from water. The oxidized cellulose was then filtered on a Buchner funnel and washed with deionized water until neutrality and then titrated for the estimation of the obtained concentration of carboxylic acids on the cellulose structure (oxidation degree). This latter was performed by titration of –COOH groups with NaOH 0.1 N using phenolphthalein as colorimetric indicator. The oxidation degree was estimated to be around 1.5 mmol_COOH_/g_TOCNF_.

### 4.2. Synthesis of CNS

3.5 g of TOCNF were suspended in 140 mL of deionized water, in order to obtain a 2.5% *w*/*v* solution. Granular NaOH (5.25 mmol, 0.210 g) was added, and the suspension was ultrasonicated to further promote the separation of the nanofibers, obtaining a transparent mixture. This latter was then acidified with 1 N HCl (20 mL), filtered and washed until neutral pH. The residual water content was calculated and the remaining water necessary to obtain a 2.8–3% *w*/*v* TOCNF solution was split up in three quotas, in order to re-suspend the cellulose and dissolve the cross-linking polymer (bPEI 25 kDa) and the co-reticulant agent CA, in three separate batches. The amount of the cross-linking polymer (7 g) was calculated as double of the initial weight of TOCNF and 1.8 g of co-reticulant were used. Once dissolved in water, the cross-linker and the co-reticulant agents were slowly added to the TOCNF solution, while continuously stirring up to obtain a white and homogeneous hydrogel, which was placed in 24-wells well-plates and quickly frozen at −35 °C. After 24 h, the well-plates were moved to the freeze-dryer. At the end of the process, white cylindrical-shaped spongy aerogels were obtained. They were removed from the wells and placed in an oven, at the initial temperature of 55 °C. The temperature was then slowly raised up to 102 °C and then kept constant for 16 h. At the end of the thermal treatment, CNS were washed 6 times with deionized water (6 × 100 mL) and the last time with 97% ethanol solution (1 × 50 mL).

### 4.3. Metal Loading on CNS

The uploading of the metals on CNS’s bulk was carried out with aqueous solutions of Zn(NO_3_)_2_ (prepared dissolving 250 g of Zn(NO_3_)_2_ in 250 mL deionized water) and CuSO_4_ (prepared dissolving 80 g of CuSO_4_ in 250 mL deionized water). 1 g of CNS was stirred for 4 h in 250 mL of Zn (II) and Cu (II) solutions. At the end of the sorption process, CNS were filtered off and then washed once with deionized water (500 mL), obtaining Zn-loaded (CNS-Zn) and Cu-loaded (CNS-Cu) catalysts.

### 4.4. General Procedure for Catalytic Synthesis of Acetals

The catalyst (CNS-Zn or CNS-Cu), the reagents and finally the alcohol as solvent were added in a 5 mL microwave vial, equipped with a magnetic stirrer, in the quantities reported in Table 3, Table 4, Table 5, Table 6, Table 7, Table 8, Table 9, Table 10, Table 11 and Table 12. The reactions were conducted in a microwave reactor in a time ranging from 2 to 6 h, with a temperature comprised between 40 °C and 100 °C, at atmospheric pressure.

### 4.5. Products Characterization

All conversion and selectivity data were calculated by ^1^H-NMR analysis on the crude (see Supplementary Materials). For a better accuracy, the percentage of the acid by-product in the mixture, often present in very low amounts, was determined by GC-MS analysis.

The ^1^H-NMR spectra of known products **1**, **3a–i** and **7a–h**, are in agreement with the literature references as reported in the Supplementary Materials.

Product **2** was recovered and purified according to the following procedure in order to demine the isolated yield and to compare that with the value of calculated by ^1^H-NMR with internal standard. The catalyst was removed by filtration and the alcoholic solvent was removed under vacuum. The obtained crude was dissolved in 15 mL of ethyl acetate and washed with deionized water (3 × 15 mL). The extracted organic layer was dried with sodium sulfate and the solvent was removed under vacuum by means of a Rotavapor^®^. Flash column chromatography was performed to purify the product by using 10–12 mm silica gel packing. The eluent was chosen in order to move the desired components to *R*_f_ 0.35 on analytical TLC. The selected eluent was a mixture of hexane and ethyl acetate in a 9:1 ratio.

The unknown product **5** was isolated according to procedure previously reported for product **2**. Flash column chromatography was performed to purify product **5** by using 10–12 mm silica gel packing and the selected eluent was a mixture of hexane and ethyl acetate in a 95:5 ratio. The characterization of new compound **5** is here reported.

1-(diethoxymethyl)-4-fluorobenzene **5.** ^1^H NMR (400 MHz, Chloroform-*d*) δ 7.45 (dd, *J* = 8.6, 5.6 Hz, 2H), 7.03 (t, *J* = 8.8 Hz, 2H), 5.48 (s, 1H), 3.60 (dq, *J* = 9.8, 7.2 Hz, 2H), 3.52 (dq, *J* = 9.5, 7.1 Hz, 2H), 1.23 (t, *J* = 7.1 Hz, 6H). ^13^C NMR (101 MHz, Chloroform-*d*) δ 162.7 (d, *J* = 246.2 Hz), 135.1 (d, *J* = 3.2 Hz), 128.4 (d, *J* = 8.1 Hz, 2C), 114.9 (d, *J* = 21.4 Hz, 2C), 100.9, 60.9, 15.1. Elemental analysis for C_11_H_15_FO_2_: C, 66.65%; H, 7.63%; F, 9.58%; O, 16.14%. Found C, 66.12%; H, 7.65%.

## Figures and Tables

**Table 1:** ICP-OES analysis result for the sample before the reactions. I, II and III indicate three different samples on which measurements were performed.

**CNS-Zn**					
	**I**	**II**	**III**	**Average**	**Std. Deviation**
**Zn (w%)**	6.85	6.69	6.78	6.77	0.08
**CNS-Cu**					
	**I**	**II**	**III**	**Average**	**Std. Deviation**
**Cu (w%)**	7.44	7.67	7.56	7.56	0.12

**Table 2:** ICP-OES analysis results obtained analyzing the methanol solution after a simulated standard synthetic protocol (2 h, methanol, microwave irradiation, 40 °C). For CNS-Cu 6 mg of catalyst in 15 mL of methanol were used, while for CNS-Zn 45 mg of catalyst in 15 mL of methanol were used (quantities selected according to the optimization tests discussed in Section 3).

	Cu [mg/L]	Zn [mg/L]
**Test with CNS-Zn**	0.0545 ± 0.002	0.4613 ± 0.010
**Test with CNS-Cu**	0.0515 ± 0.002	0.0883 ± 0.002

**Table 3:** Optimization of the reaction temperature in methanol ^a^.

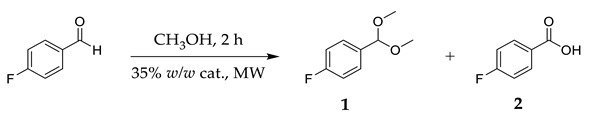					
Entry	T [°C]	Catalyst Type	% Conversion	Selectivity	
				% 1	% 2
*1*	40	CNS	-	-	-
*2*	40	CNS-Cu	42	96	4
*3*	40	CNS-Zn	98	97	3
*4*	80	CNS	-	-	-
*5*	80	CNS-Cu	92	90	10
*6*	80	CNS-Zn	96	76	24

^a^ 50 mg of *p*-F-benzaldehyde in 2.5 mL of methanol were used, processing the reaction under microwave (MW) irradiation.

**Table 4:** Optimization of the % *w*/*w* of CNS-Cu and CNS-Zn ^a^.

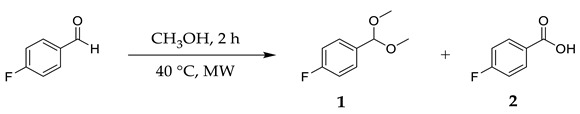					
Entry	Catalyst	% *w*/*w* Catalyst	% Conversion	Selectivity	
				% 1	% 2
*1*	CNS-Cu	35	42	96	4
*2*	CNS-Cu	15	47	94	6
*3*	CNS-Cu	2	89	96	4
*4*	CNS-Zn	35	98	97	3
*5*	CNS-Zn	15	99	96	4
*6*	CNS-Zn	2	72	95	5

^a^ 50 mg of *p*-F-benzaldehyde in 2.5 mL of methanol were used, processing the reaction in MW.

**Table 5:** Results for kinetic tests with CNS-Cu ^a^.

Time [min]	% Conversion
30	81
60	81
90	88
120	84

^a^ The reaction was conducted using 50 mg of *p*-F-benzaldehyde in 2.5 mL of methanol processing the reaction in MW, with a reaction temperature of 40 °C and a 2% *w*/*w* of CNS-Cu.

**Table 6:** Results for kinetic tests with CNS-Zn ^a^.

Time [min]	% Conversion
30	61
60	71
90	81
120	84

^a^ The reaction was conducted using 50 mg of *p*-F-benzaldehyde in 2.5 mL of methanol processing the reaction in MW, with a reaction temperature of 40 °C and a 2% *w*/*w* of CNS-Zn.

**Table 7:** Reactions with different carbonyl compounds using methanol as solvent *.

Entry	Reagent	Catalyst	% Conversion	Selectivity	
				% 3	% 4
*1*	*p*-F-benzaldehyde	CNS-Cu	74	**1**: 93	**2**: 7
*2*	*p*-Me-benzaldehyde (**a**)	CNS-Cu	94	**3a**: 98	**4a**: 2
*3*	*m*-OMe-benzaldehyde (**b**)	CNS-Cu	69	**3b**: 100	**4b**: -
*4*	*o*-OMe-benzaldehyde (**c**)	CNS-Cu	93	**3c**: 98	**4c**: 2
*5*	Cyclohexanone (**d**)	CNS-Cu	96	**3d**: 100	**4d**: -
*6*	Cyclopentanone (**e**)	CNS-Cu	55	**3e**: 100	**4e**: -
*7*	2-Naphtaldehyde (**f**)	CNS-Cu	86	**3f**: 84	**4f**: 16
*8*	2-Furaldehyde (**g**)	CNS-Cu	52	**3g**: 100	**4g**: -
*9*	Benzaldehyde (**h**)	CNS-Cu	90	**3h**: 87	**4h**: 13
*10*	*p*-Cl-benzaldehyde (**i**)	CNS-Cu	35	**3i**: 100	**4i**: -
*11*	*p*-F-benzaldehyde	CNS-Zn	94	**1**: 97	**2**: 3
*12*	*p*-Me-benzaldehyde (**a**)	CNS-Zn	98	**3a**: 100	**4a**: -
*13*	*m*-OMe-benzaldehyde (**b**)	CNS-Zn	97	**3b**: 100	**4b**: -
*14*	*o*-OMe-benzaldehyde (**c**)	CNS-Zn	99	**3c**: 98	**4c**: 2
*15*	Cyclohexanone (**d**)	CNS-Zn	100	**3d**: 100	**4d**: -
*16*	Cyclopentanone (**e**)	CNS-Zn	55	**3e**: 100	**4e**: -
*17*	2-Naphtaldehyde (**f**)	CNS-Zn	87	**3f**: 98	**4f**: 2
*18*	2-Furaldehyde (**g**)	CNS-Zn	49	**3g**: 100	**4g**: -
*19*	Benzaldehyde (**h**)	CNS-Zn	98	**3h**: 100	**4h**: -
*20*	*p*-Cl-benzaldehyde (**i**)	CNS-Zn	37	**3i**: 00	**4i**: -

* All reactions were conducted in MW at 40 °C for 2 h, using 50 mg of carbonyl compound and 2.5 mL of methanol, with 2% *w*/*w* percentage for CNS-Cu and 15% *w*/*w* percentage for CNS-Zn.

**Table 8:** Comparison between the same reaction conditions in methanol and in ethanol ^a^.

Solvent	% Conversion	Selectivity (%)	
		% Acetal	% Acid
Methanol	90	1: 87	2: 13
Ethanol	33	5: 63 ^b^	2: 37

^a^ The reaction was conducted using 50 mg of *p*-F-benzaldehyde in 2.5 mL of solvent processing the reaction in MW, with a reaction temperature of 40 °C, a reaction time of 2 h and a 2% *w*/*w* of CNS-Cu. ^b^ Product **5** is reported in the scheme of Table 9.

**Table 9:** Optimization of the reaction conditions using ethanol as solvent ^a^.

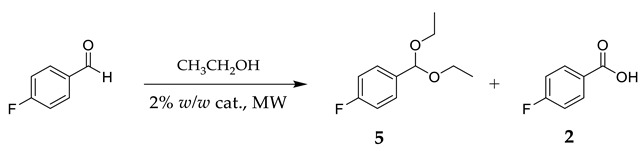					
Entry	T [°C]	t [h]	% Conversion	Selectivity	
				% 5	% 2
*1*	80	2	61	78	22
*2*	80	4	75	82	18
*3*	80	6	86	82	18
*4*	100	2	68	79	21
*5*	100	4	87	80	20
*6*	100	6	85	81	19

^a^ 50 mg of *p*-F-benzaldehyde in 2.5 mL of ethanol were used, processing the reaction in MW. Catalyst type: CNS-Cu.

**Table 10:** Reactions with different carbonyl compounds using ethanol as solvent *.

Entry	Reagent	Catalyst	% Conversion	Selectivity	
				% 6	% 4
*1*	*p*-F-benzaldehyde	CNS-Cu	68	**5**: 97	**2**: 3
*2*	*p*-Me-benzaldehyde (**a**)	CNS-Cu	50	**6a**: 92	**4a**: 8
*3*	*m*-OMe-benzaldehyde (**b**)	CNS-Cu	12	**6b**: 100	**4b**: -
*4*	*o*-OMe-benzaldehyde (c)	CNS-Cu	70	**6c**: 98	**4c**: 2
*5*	Cyclohexanone (**d**)	CNS-Cu	48	**6d**: 100	**4d**: -
*6*	Cyclopentanone (**e**)	CNS-Cu	24	**6e**: 100	**4e**: -
*7*	2-Furaldehyde (**g**)	CNS-Cu	31	**6g**: 100	**4g**: -
*8*	Benzaldehyde (**h**)	CNS-Cu	87	**6h**: 80	**4h**: 20
*9*	*p*-Cl-benzaldehyde (**i**)	CNS-Cu	2	**6i**: 100	**4i**: -
*10*	*p*-F-benzaldehyde	CNS-Zn	84	**5**: 97	**2**: 3
*11*	*p*-Me-benzaldehyde (**a**)	CNS-Zn	76	**6a**: 97	**4a**: 3
*12*	*m*-OMe-benzaldehyde (**b**)	CNS-Zn	80	**6b**: 98	**4b**: 2
*13*	*o*-OMe-benzaldehyde (**c**)	CNS-Zn	89	**6c**: 99	**4c**: 1
*14*	Cyclohexanone (**d**)	CNS-Zn	58	**6d**: 100	**4d**: -
*15*	Cyclopentanone (**e**)	CNS-Zn	31	**6e**: 100	**4e**: -
*16*	2-Furaldehyde (**g**)	CNS-Zn	96	**6g**: 97	**4g**: 3
*17*	Benzaldehyde (**h**)	CNS-Zn	93	**6h**: 84	**4h**: 16
*18*	*p*-Cl-benzaldehyde (**i**)	CNS-Zn	84	**6i**: 100	**4i**: 0

* All reactions were conducted in MW at 100 °C for 4 h, using 50 mg of carbonyl compound and 2.5 mL of EtOH, with the catalyst at 2% *w*/*w* percentage for CNS-Cu and at 15% *w*/*w* percentage for CNS-Zn.

**Table 11:** Results of the reusability tests conducted with CNS-Cu ^a^.

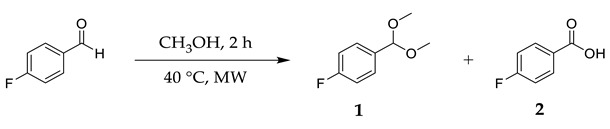					
Entry	T [°C]	t [h]	% Conversion	Selectivity	
				% 1	% 2
A	80	2	72	97	3
B	80	4	88	98	2
C	80	6	83	97	3
D	100	2	49	68	32

^a^ 50 mg of *p*-F-benzaldehyde in 2.5 mL of methanol were used, processing the reaction in MW. Catalyst type: CNS-Cu % *w*/*w* of catalyst: 2%.

**Table 12:** Results of the reusability tests conducted with CNS-Zn ^a^.

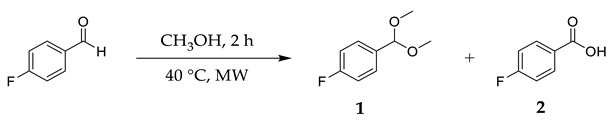					
Entry	T [°C]	t [h]	% Conversion	Selectivity	
				% 1	% 2
A	80	2	85	83	17
B	80	4	85	88	12
C	80	6	92	89	11
D	100	2	86	78	22

^a^ 50 mg of *p*-F-benzaldehyde in 2.5 mL of methanol were used, processing the reaction in MW. Catalyst type: CNS-Zn % *w*/*w* of catalyst: 15%.

## Supplementary Materials

The following supporting information can be downloaded at: https://www.mdpi.com/article/10.3390/gels8010054/s1. ^1^H NMR Spectra of the crude products for all the known compounds used to determine the product yield. Figure S1: ^1^H NMR spectrum of product **1** in CDCl_3_; Figure S2: ^1^H NMR spectrum of product **3a** in CDCl_3_; Figure S3: ^1^H NMR spectrum of product **3b** in CDCl_3_; Figure S4: ^1^H NMR spectrum of product **3c** in CDCl_3_; Figure S5: ^1^H NMR spectrum of product **3d** in CDCl_3_; Figure S6: ^1^H NMR spectrum of product **3e** in CDCl_3_; Figure S7: ^1^H NMR spectrum of product **3f** in CDCl_3_; Figure S8: ^1^H NMR spectrum of product **3g** in CDCl_3_; Figure S9: ^1^H NMR spectrum of product **3h** in CDCl_3_; Figure S10: ^1^H NMR spectrum of product **3i** in CDCl_3_; Figure S11: ^1^H NMR spectrum of product **6a** in CDCl_3_; Figure S12: ^1^H NMR spectrum of product **6b** in CDCl_3_; Figure S13: ^1^H NMR spectrum of product **6c** in CDCl_3_; Figure S14: ^1^H NMR spectrum of product **6d** in CDCl_3_; Figure S15: ^1^H NMR spectrum of product **6e** in CDCl_3_; Figure S16: ^1^H NMR spectrum of product **6g** in CDCl_3_; Figure S17: ^1^H NMR spectrum of product **6h** in CDCl_3_; Figure S18: ^1^H NMR spectrum of product **6i** in CDCl_3_; Figure S19: ^1^H NMR spectrum of product **5** in CDCl_3_; Figure S20: ^13^C-APT NMR spectrum of product **5** in CDCl_3_; Figure S21: ^13^C NMR spectrum of product **5** in CDCl_3_; Figure S22: COSY NMR spectrum of product **5** in CDCl_3_; Figure S23: HSQC NMR spectrum of product **5** in CDCl_3_.
