# Supplementary material for: Zinc- and Copper-Loaded Nanosponges from Cellulose Nanofibers Hydrogels: New Heterogeneous Catalysts for the Synthesis of Aromatic Acetals
Source: Gels. 2022 Jan 12;8(1):54. doi: 10.3390/gels8010054 (PMC8774417; doi:10.3390/gels8010054)
Supplement: Supplementary file 1 [file gels-08-00054-s001.zip › gels-1545120-supplementary.pdf]

gels

Supplementary

Zinc- and Copper-Loaded Nanosponges from Cellulose Nanofibers Hydrogels: New Heterogeneous Catalysts for the Synthesis of Aromatic Acetals

Laura Riva, Angelo Davide Lotito, Carlo Punta, and Alessandro Sacchetti *

Department of Chemistry, Materials, and Chemical Engineering "G. Natta" and INSTM Local Unit, Politecnico di Milano, 20131 Milan, Italy; laura2.riva@polimi.it (L.R.); angelodavide.lotito@mail.polimi.it (A.D.L.); carlo.punta@polimi.it (C.P.)
* Correspondence: alessandro.sacchetti@polimi.it; Tel.: +39-0223993017

Citation: Riva, L.; Lotito, A.D.; Punta, C.; Sacchetti, A. Zinc- and Copper-Loaded Nanosponges from Cellulose Nanofibers Hydrogels: New Heterogeneous Catalysts for the Synthesis of Aromatic Acetals. *Gels* **2022**, *8*, 54. https://doi.org/10.3390/gels8010054

Academic Editors: Yi Cao

Received: 22 December 2021
Accepted: 10 January 2022
Published: 12 January 2022

Publisher's Note: MDPI stays neutral with regard to jurisdictional claims in published maps and institutional affiliations.

Copyright: © 2022 by the authors. Licensee MDPI, Basel, Switzerland. This article is an open access article distributed under the terms and conditions of the Creative Commons Attribution (CC BY) license (https://creativecommons.org/licenses/by/4.0/).

NMR Spectra

NMR of the crude products are here reported. All the spectra were recorded with a 400 MHz Brüker NMR spectrometer. Products have not been purified. The ^{1}H-NMR characterization of all products is in agreement with the literature (see references herein reported). Full NMR characterization of new compound **5** is here reported.

Figure S1: ^{1}H NMR spectrum of 1-(dimethoxymethyl)-4-fluorobenzene **1** p2
Figure S2: ^{1}H NMR spectrum of 1-(dimethoxymethyl)-4-methylbenzene **3a** p2
Figure S3: ^{1}H NMR spectrum of 1-(dimethoxymethyl)-3-methoxybenzene **3b** p3
Figure S4: ^{1}H NMR spectrum of 1-(dimethoxymethyl)-2-methoxybenzene **3c** p3
Figure S5: ^{1}H NMR spectrum of 1,1-dimethoxycyclohexane **3d** p4
Figure S6: ^{1}H NMR spectrum of 1,1-dimethoxycyclopentane **3e** p4
Figure S7: ^{1}H NMR spectrum of 2-(dimethoxymethyl)naphthalene **3f** p5
Figure S8: ^{1}H NMR spectrum of 2-(dimethoxymethyl)furan **3g** p5
Figure S9: ^{1}H NMR spectrum of (dimethoxymethyl)benzene **3h** p6
Figure S10: ^{1}H NMR spectrum of 1-chloro-4-(dimethoxymethyl)benzene **3i** p6
Figure S11: ^{1}H NMR spectrum of 1-(diethoxymethyl)-4-methylbenzene **6a** p7
Figure S12: ^{1}H NMR spectrum of 1-(diethoxymethyl)-3-methoxybenzene **6b** p7
Figure S13: ^{1}H NMR spectrum of 1-(diethoxymethyl)-2-methoxybenzene **6c** p8
Figure S14: ^{1}H NMR spectrum of 1,1-diethoxycyclohexane **6d** p8
Figure S15: ^{1}H NMR spectrum of 1,1-diethoxycyclopentane **6e** p9
Figure S16: ^{1}H NMR spectrum of 2-(diethoxymethyl)furan **6g** p9
Figure S17: ^{1}H NMR spectrum of (diethoxymethyl)benzene **6h** p10
Figure S18: ^{1}H NMR spectrum of 1-chloro-4-(diethoxymethyl)benzene **6i** p10
Figure S19: ^{1}H NMR spectrum of 1-(diethoxymethyl)-4-fluorobenzene **5** p11
Figure S20: ^{13}C-APT NMR spectrum of 1-(diethoxymethyl)-4-fluorobenzene **5** p11
Figure S21: ^{13}C NMR spectrum of 1-(diethoxymethyl)-4-fluorobenzene **5** p12
Figure S22: COSY NMR spectrum of 1-(diethoxymethyl)-4-fluorobenzene **5** p12
Figure S23: HSQC NMR spectrum of 1-(diethoxymethyl)-4-fluorobenzene **5** p13

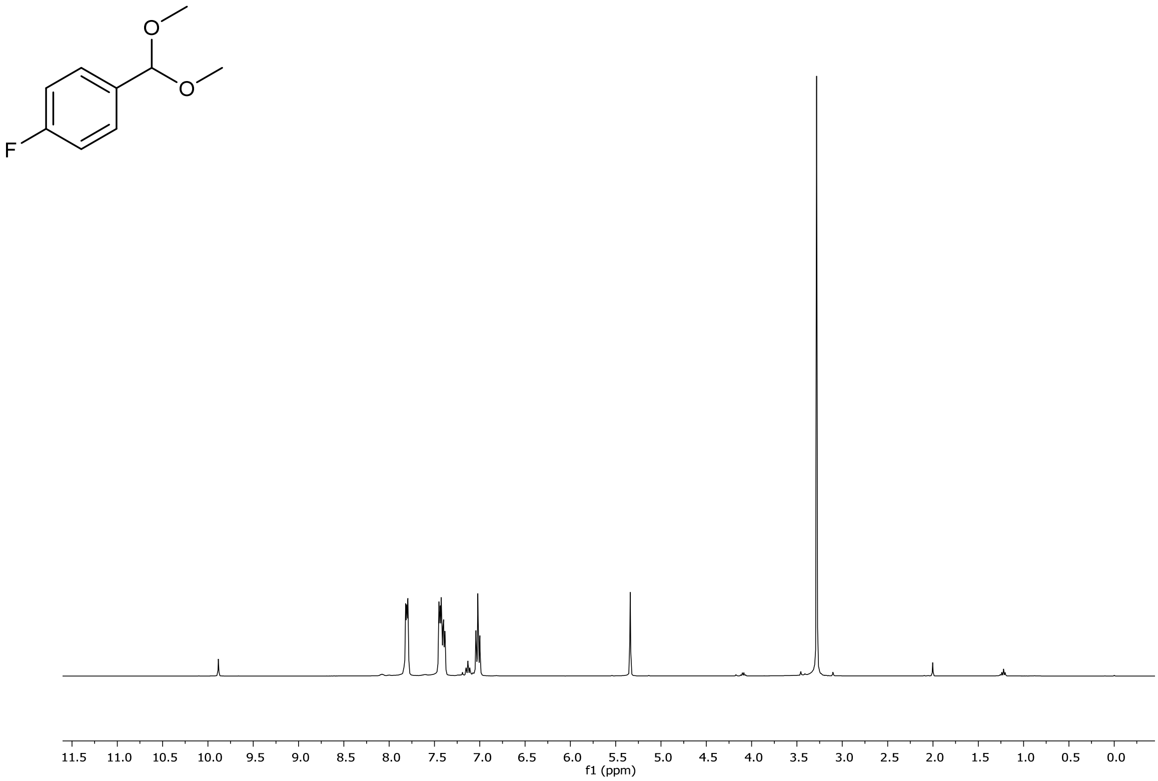

Figure S1. ^{1}H NMR spectrum of product **1** in $CDCl_3$ [1].

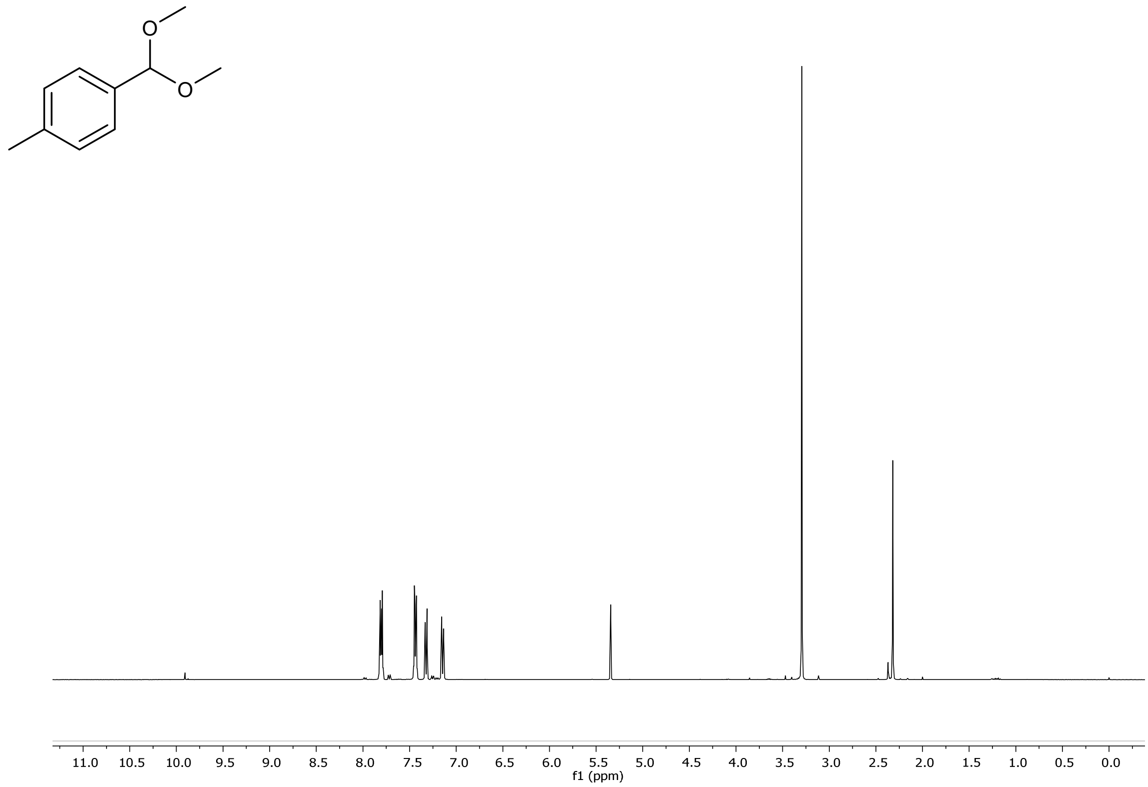

Figure S2. ^{1}H NMR spectrum of product **3a** in $CDCl_3$ [1].

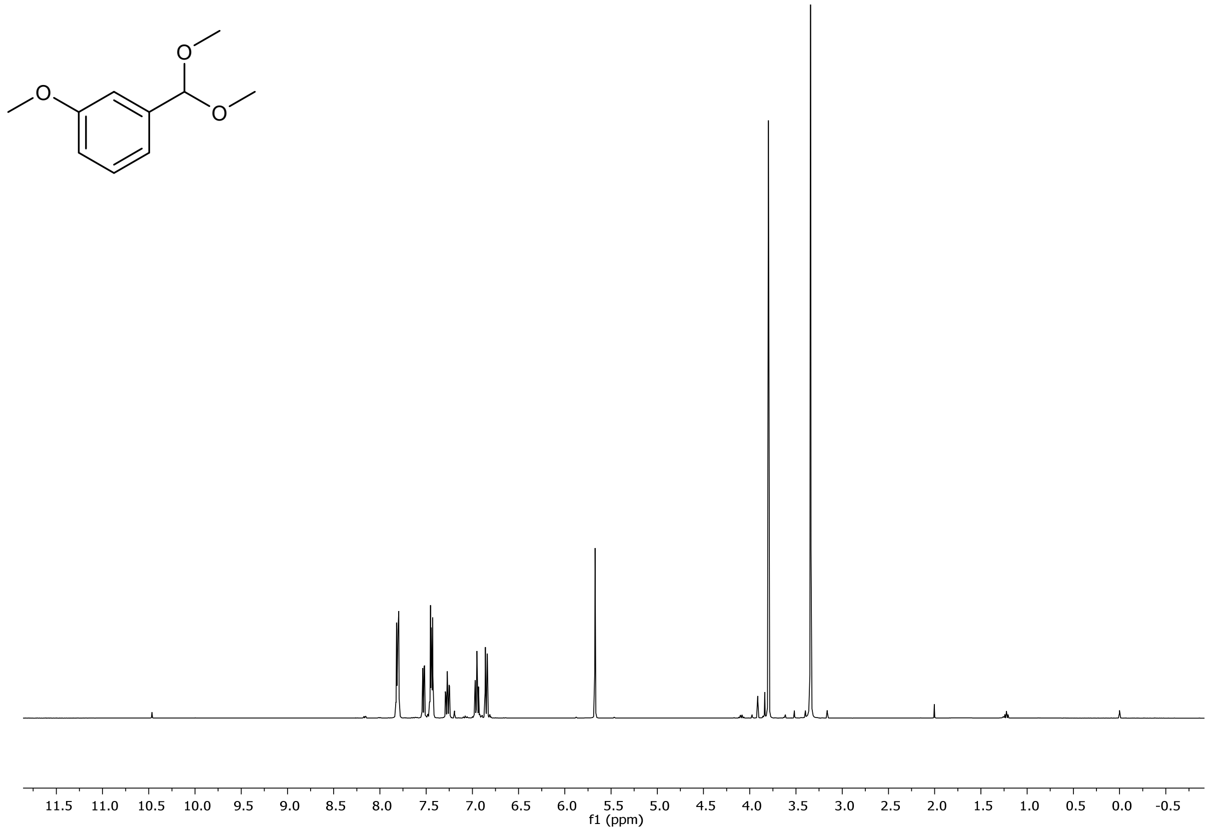

Figure S3. ^{1}H NMR spectrum of product **3b** in $CDCl_3$ [2].

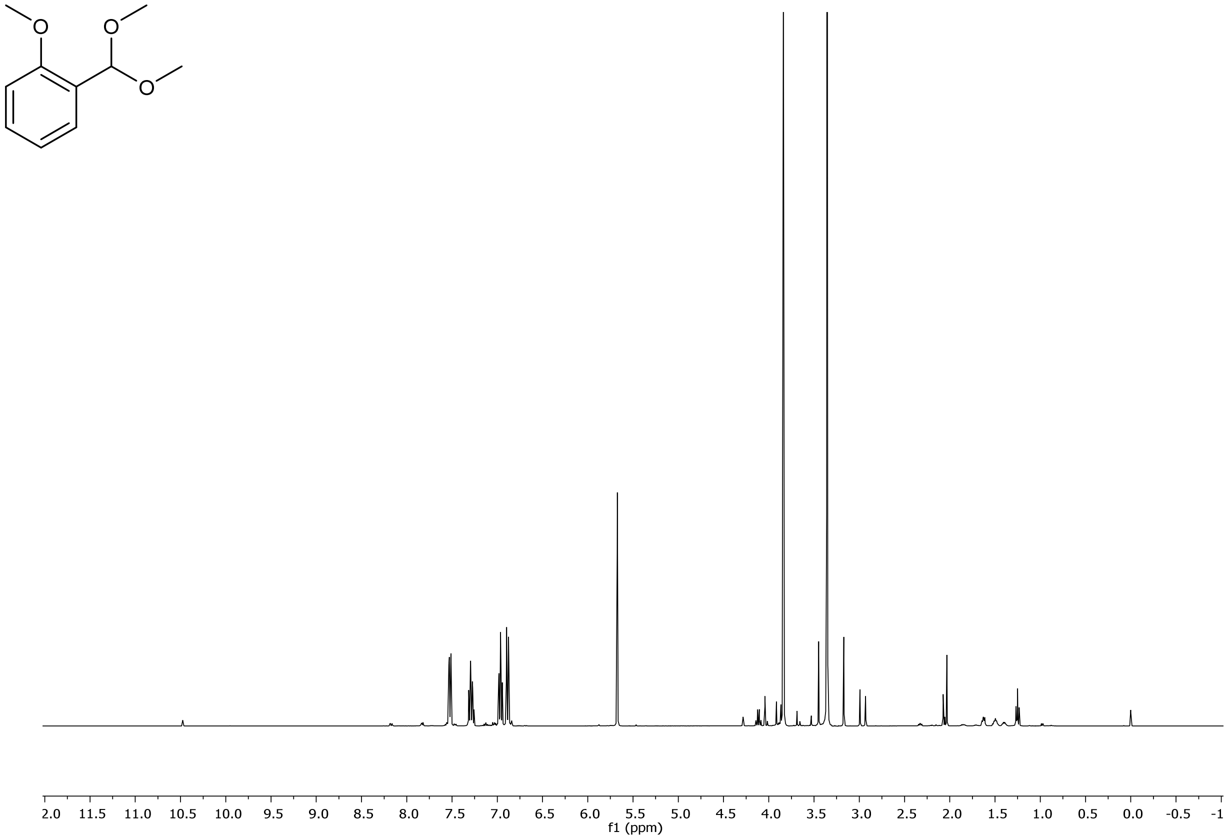

Figure S4. ^{1}H NMR spectrum of product **3c** in $CDCl_3$ [3].

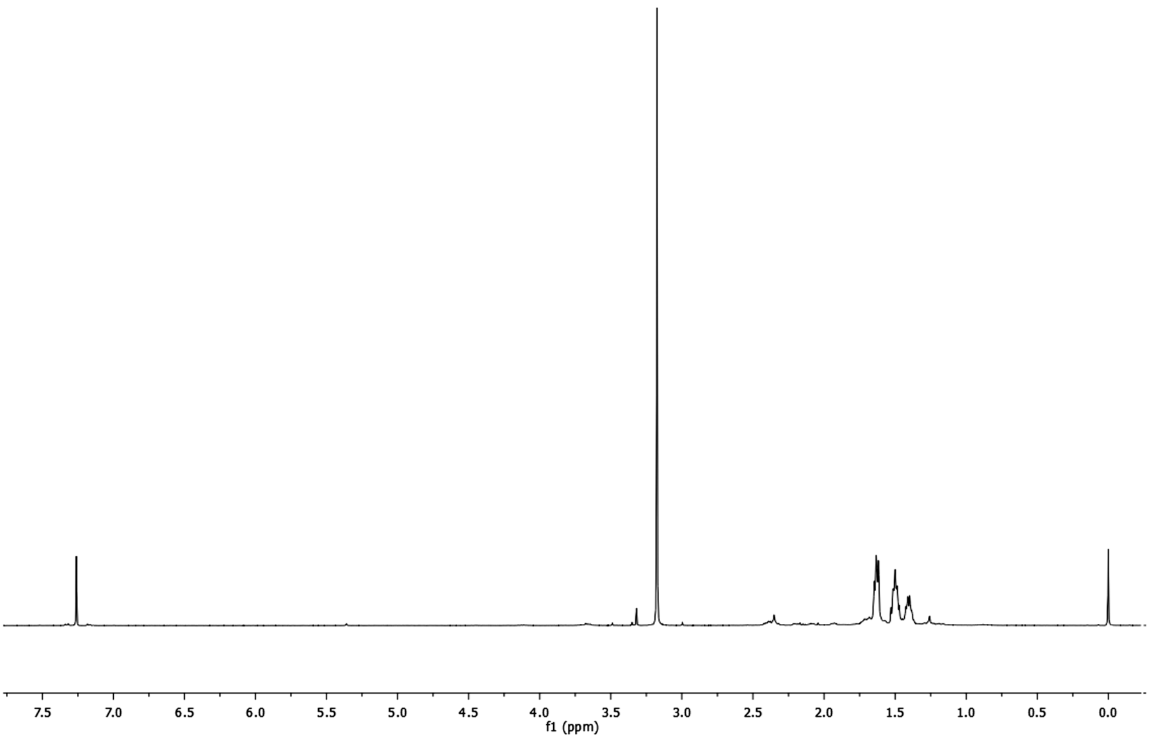

Figure S5. ^{1}H NMR spectrum of product **3d** in $CDCl_3$ [4].

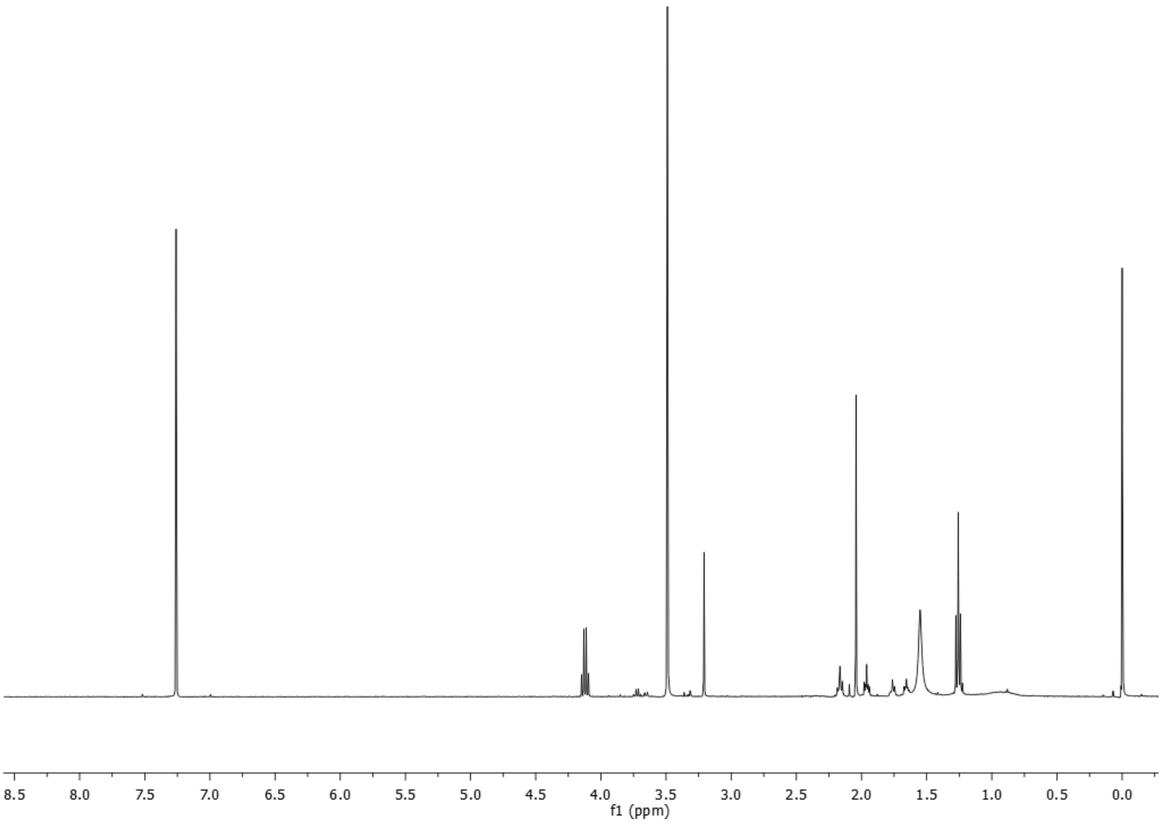

Figure S6. ^{1}H NMR spectrum of product **3e** in $CDCl_3$ [5].

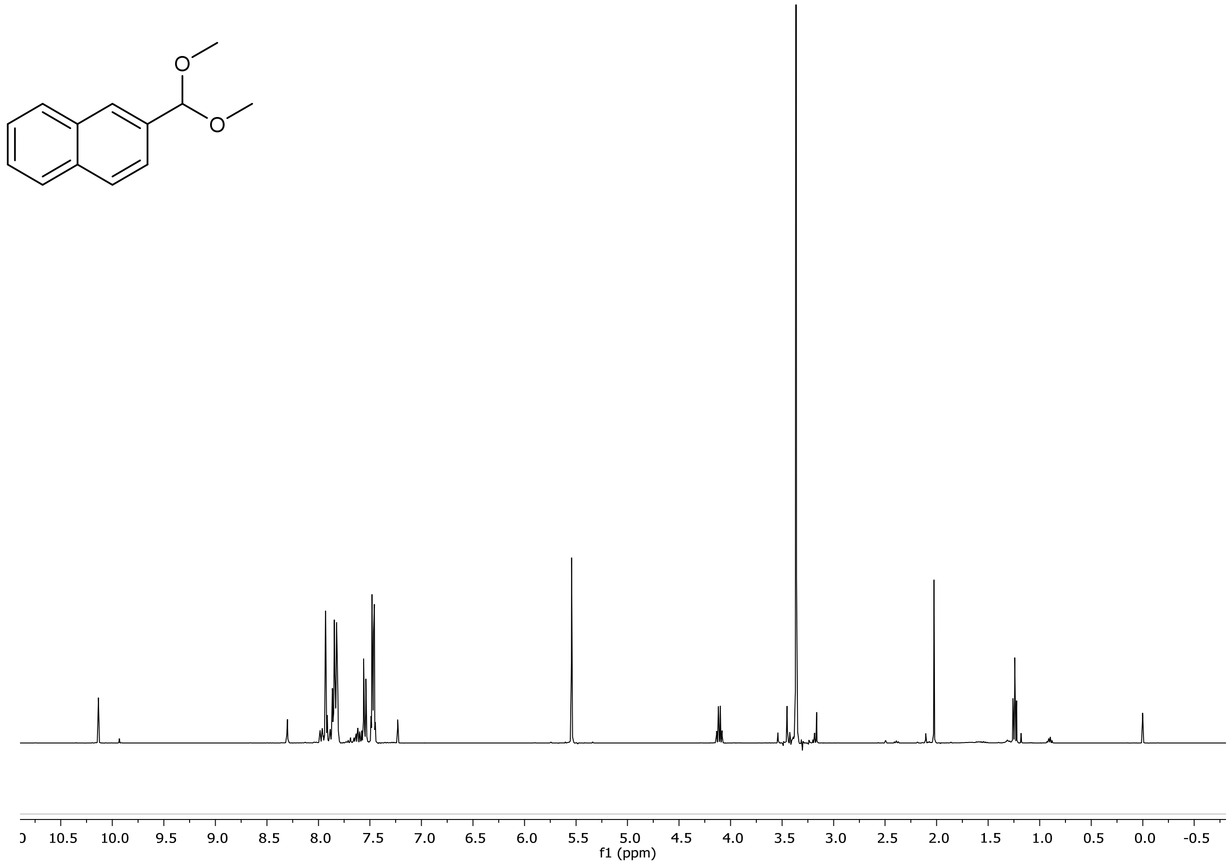

Figure S7. ^{1}H NMR spectrum of product **3f** in $CDCl_3$ [6].

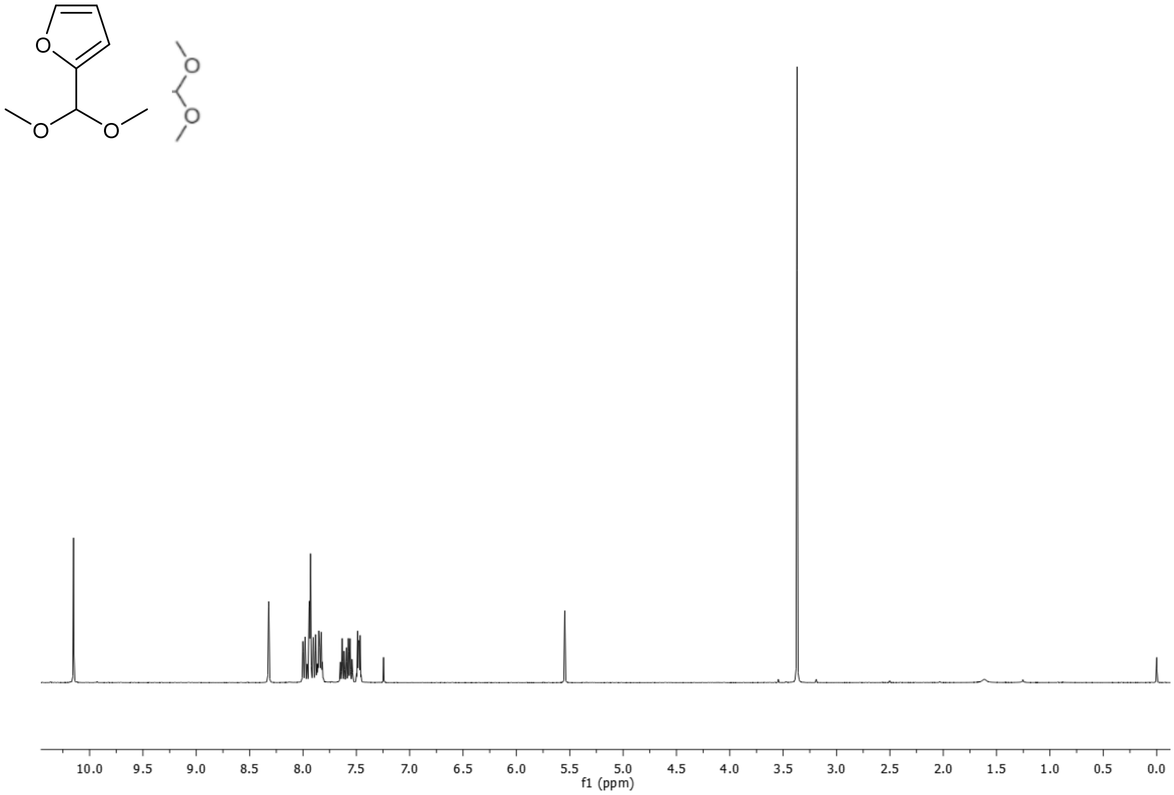

Figure S8. ^{1}H NMR spectrum of product **3g** in $CDCl_3$ [7].

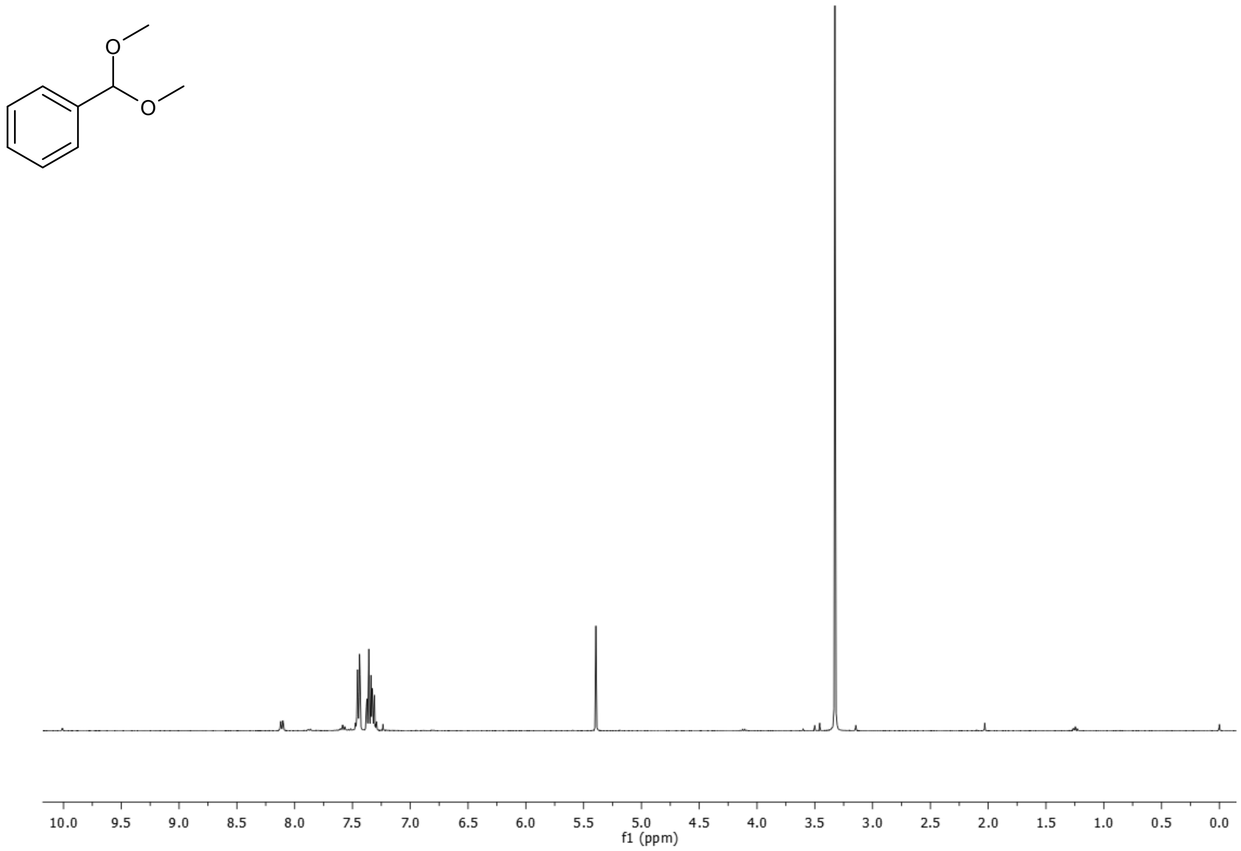

Figure S9. ^{1}H NMR spectrum of product **3h** in $CDCl_3$ [7].

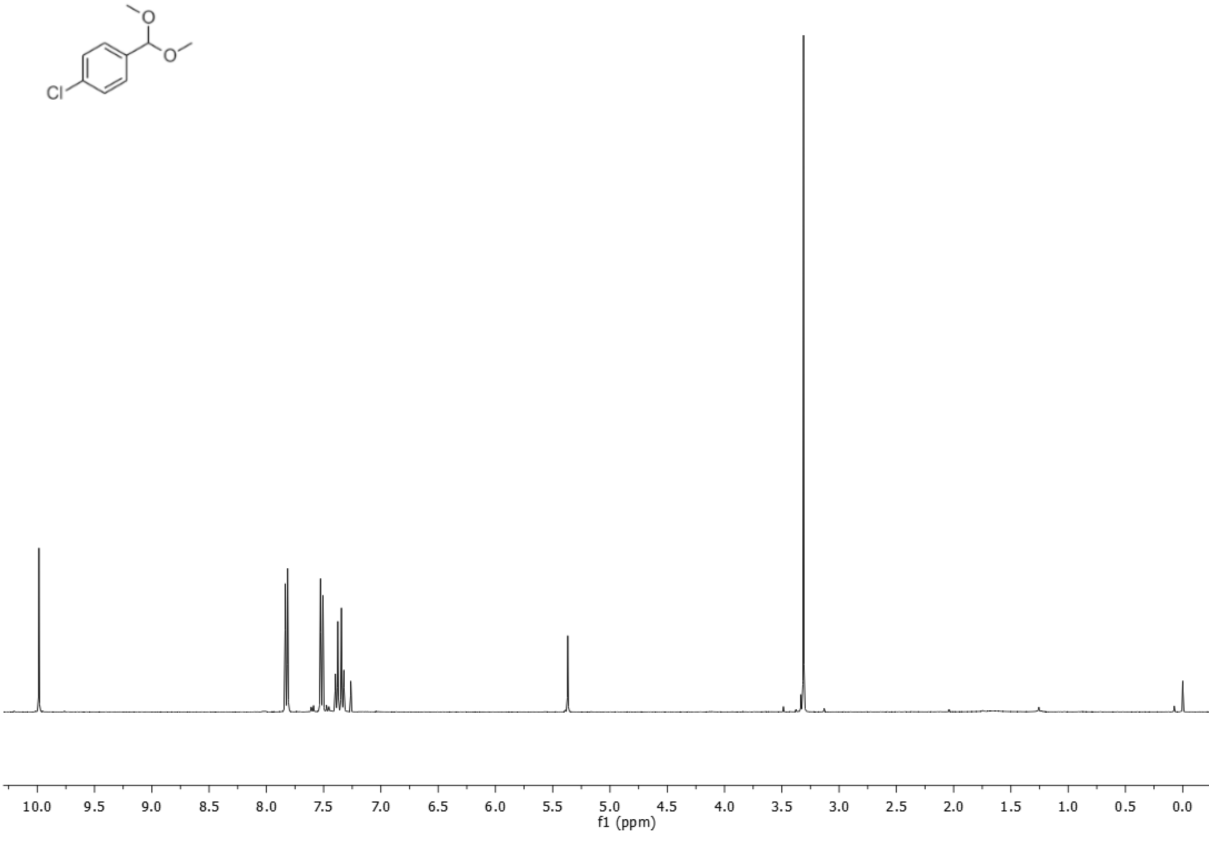

Figure S10. ^{1}H NMR spectrum of product **3i** in $CDCl_3$ [1].

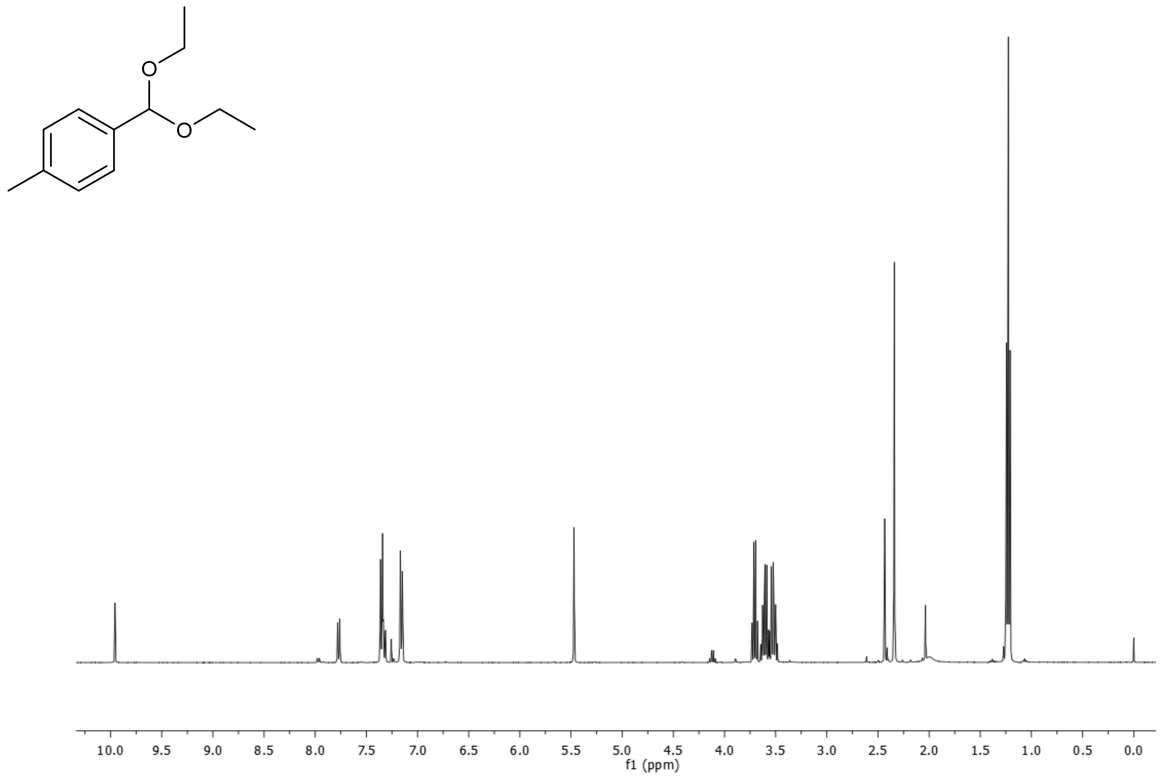

Figure S11. ^{1}H NMR spectrum of product **6a** in $CDCl_3$ [8].

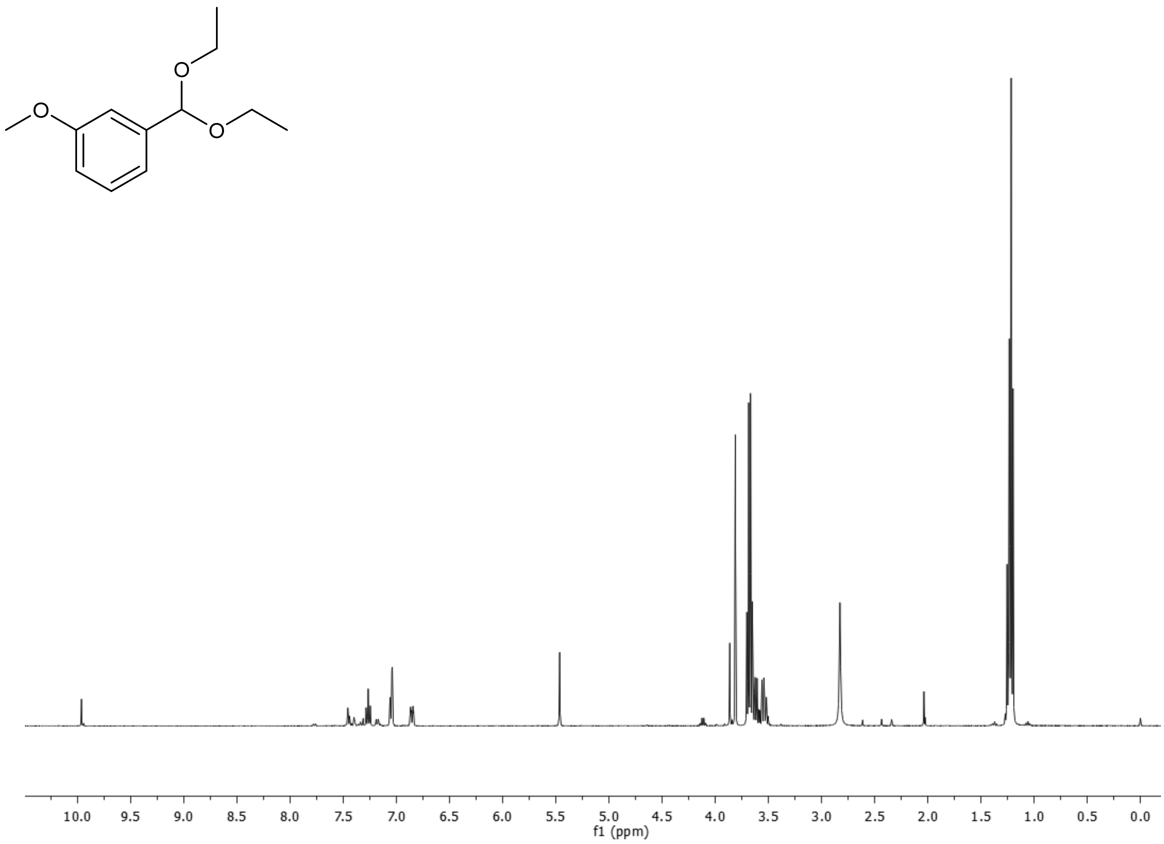

Figure S12. ^{1}H NMR spectrum of product **6b** in $CDCl_3$ [9].

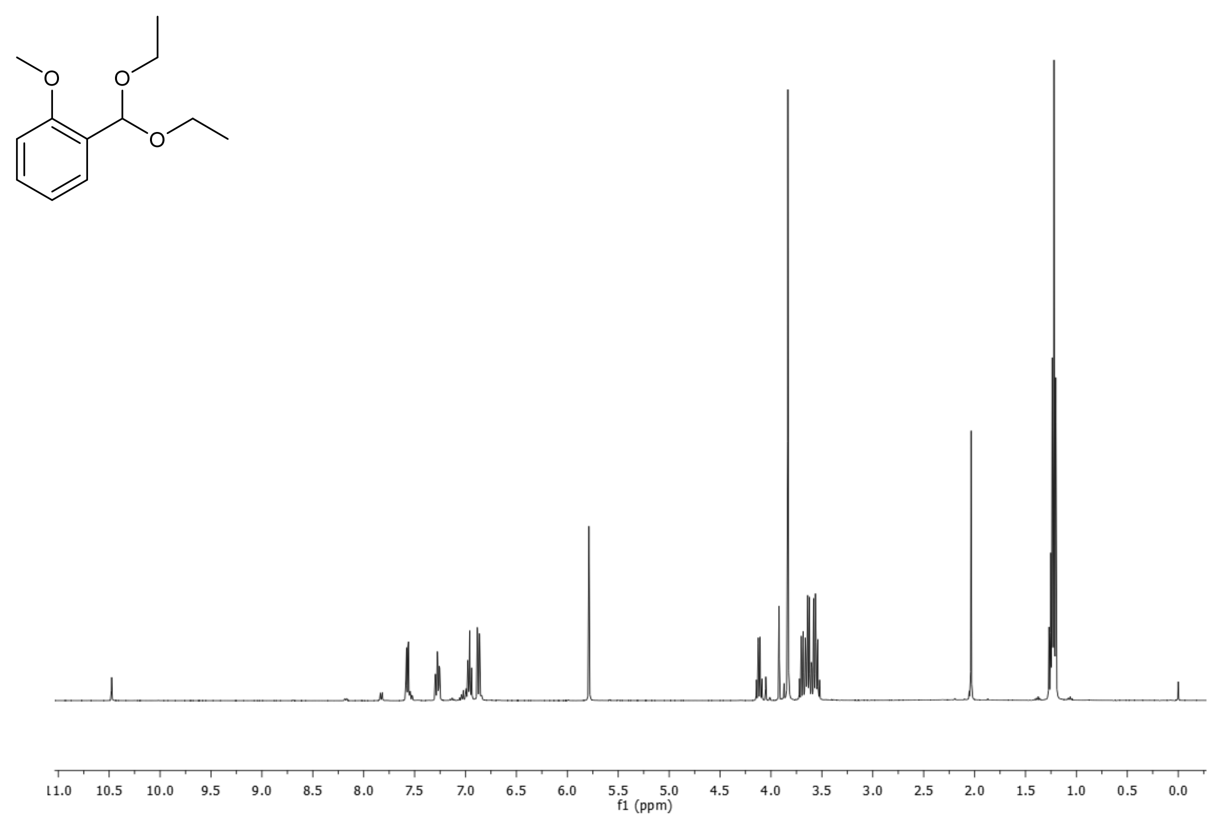

Figure S13. ^{1}H NMR spectrum of product **6c** in $CDCl_3$ [9].-

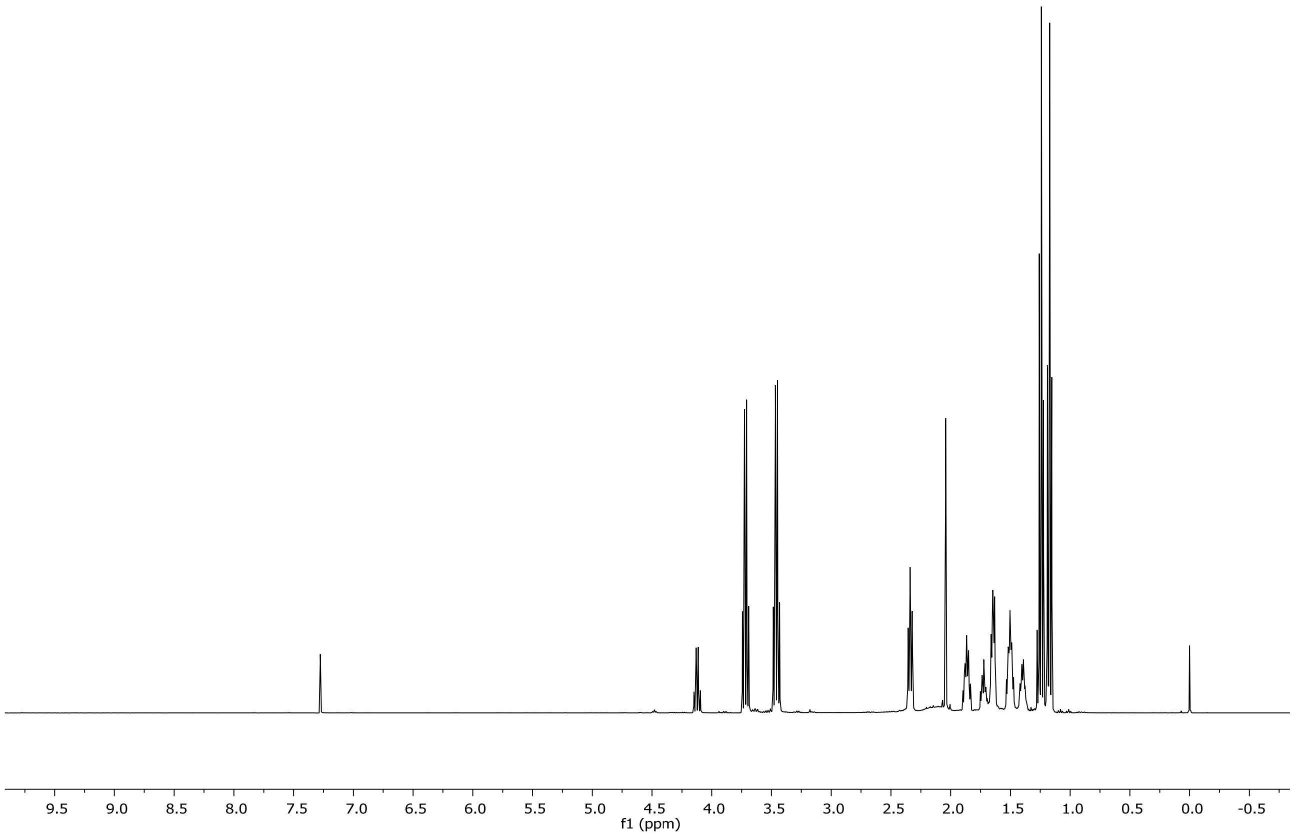

Figure S14. ^{1}H NMR spectrum of product **6d** in $CDCl_3$ [8].

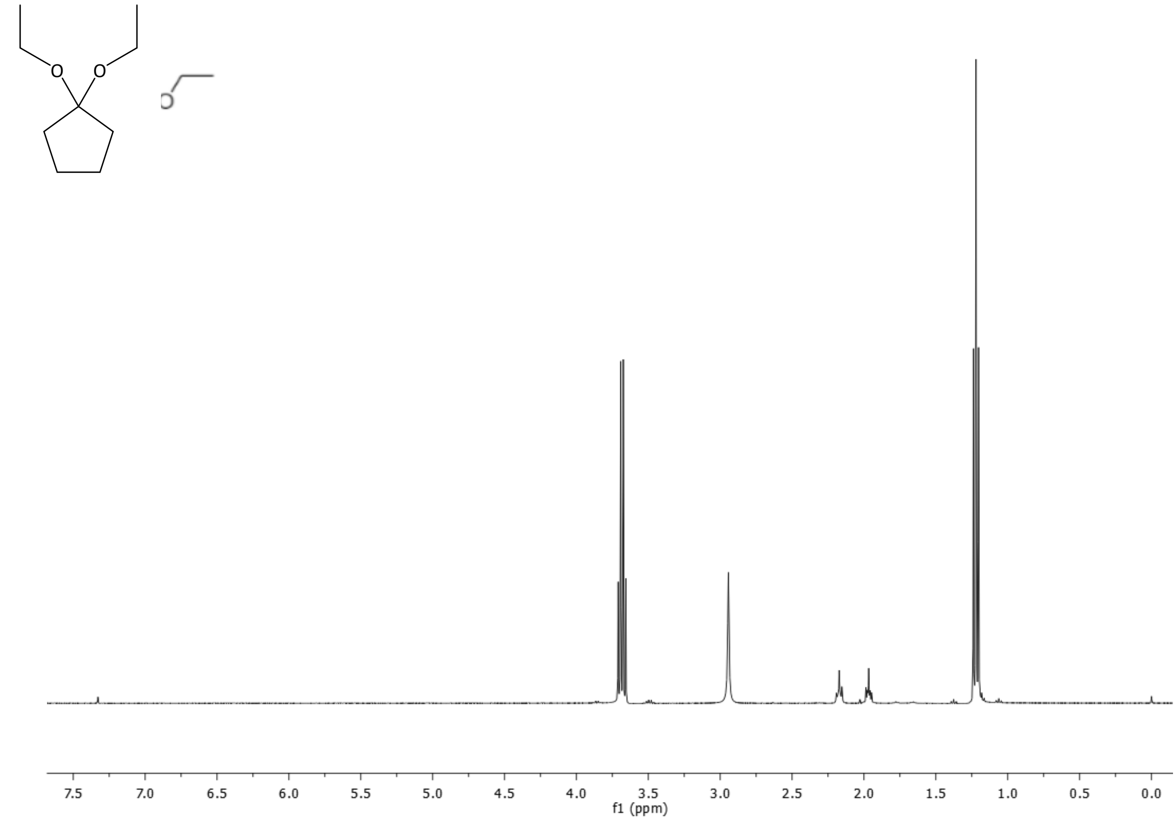

Figure S15. ^{1}H NMR spectrum of product **6e** in $CDCl_3$ [10].

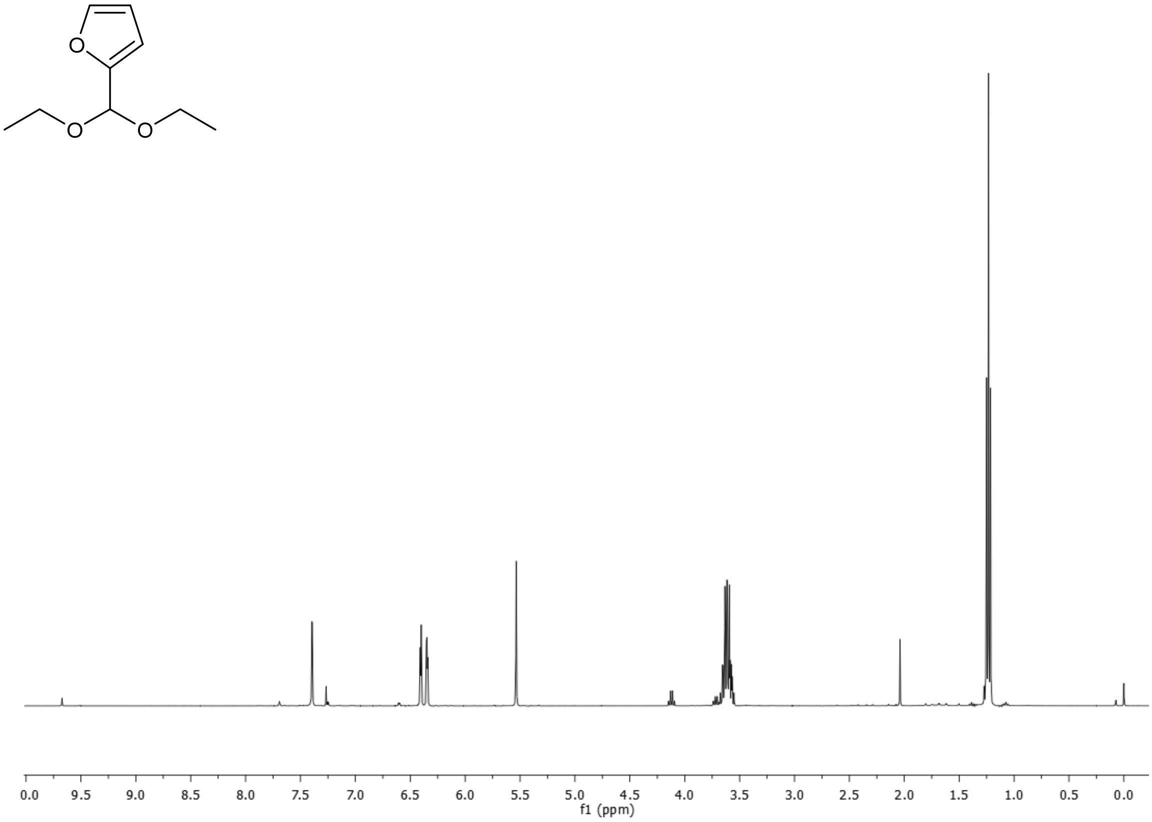

Figure S16. ^{1}H NMR spectrum of product **6g** in $CDCl_3$ [11].

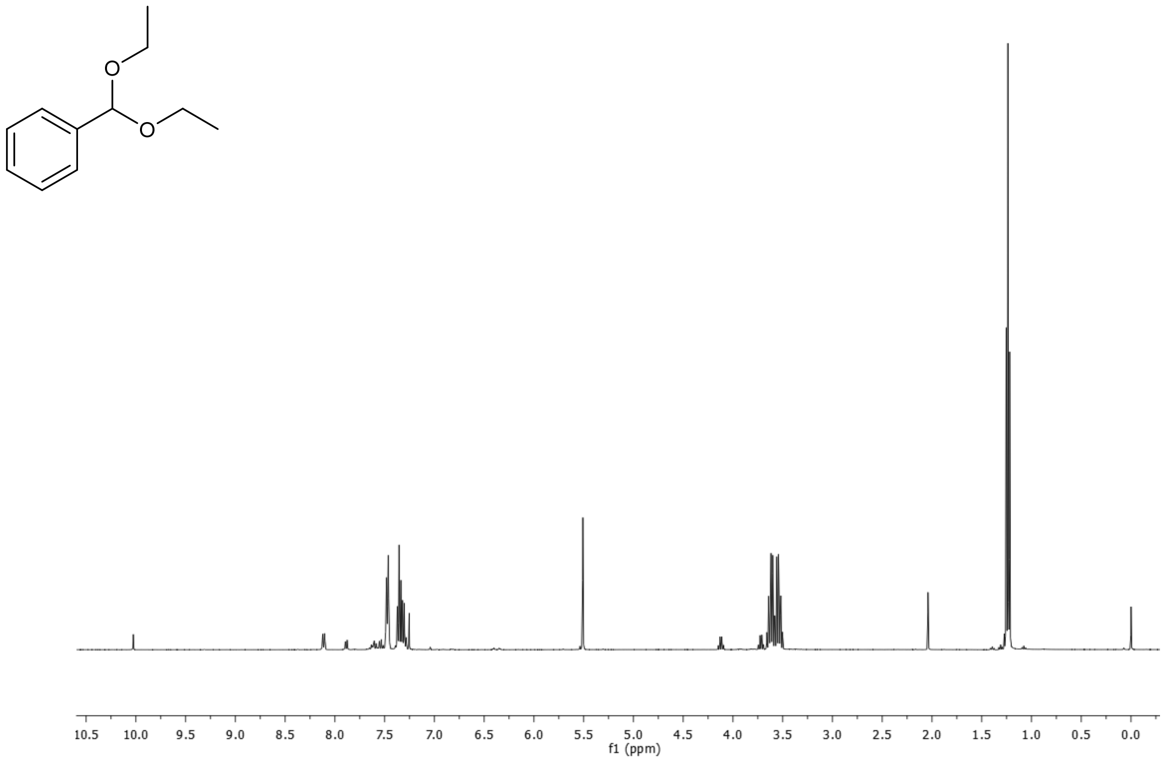

Figure S17. ^{1}H NMR spectrum of product **6h** in $CDCl_3$ [8].

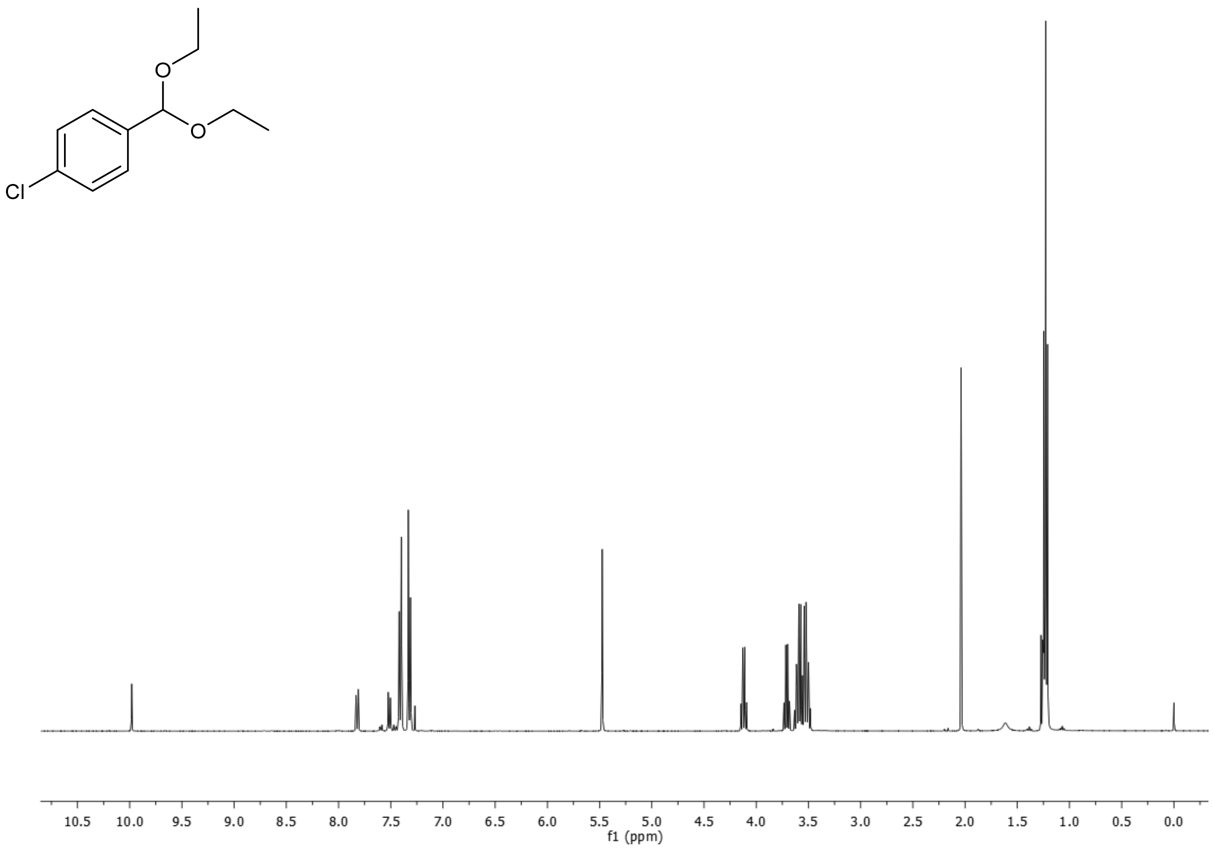

Figure S18. ^{1}H NMR spectrum of product **6i** in $CDCl_3$ [12].

The reaction product obtained reacting *p*-F-Benzaldehyde and ethanol (product **5**) is not characterized in literature. Isolation and complete characterization were carried out through NMR analysis. Below, complete NMR characterization of product **5** is reported.

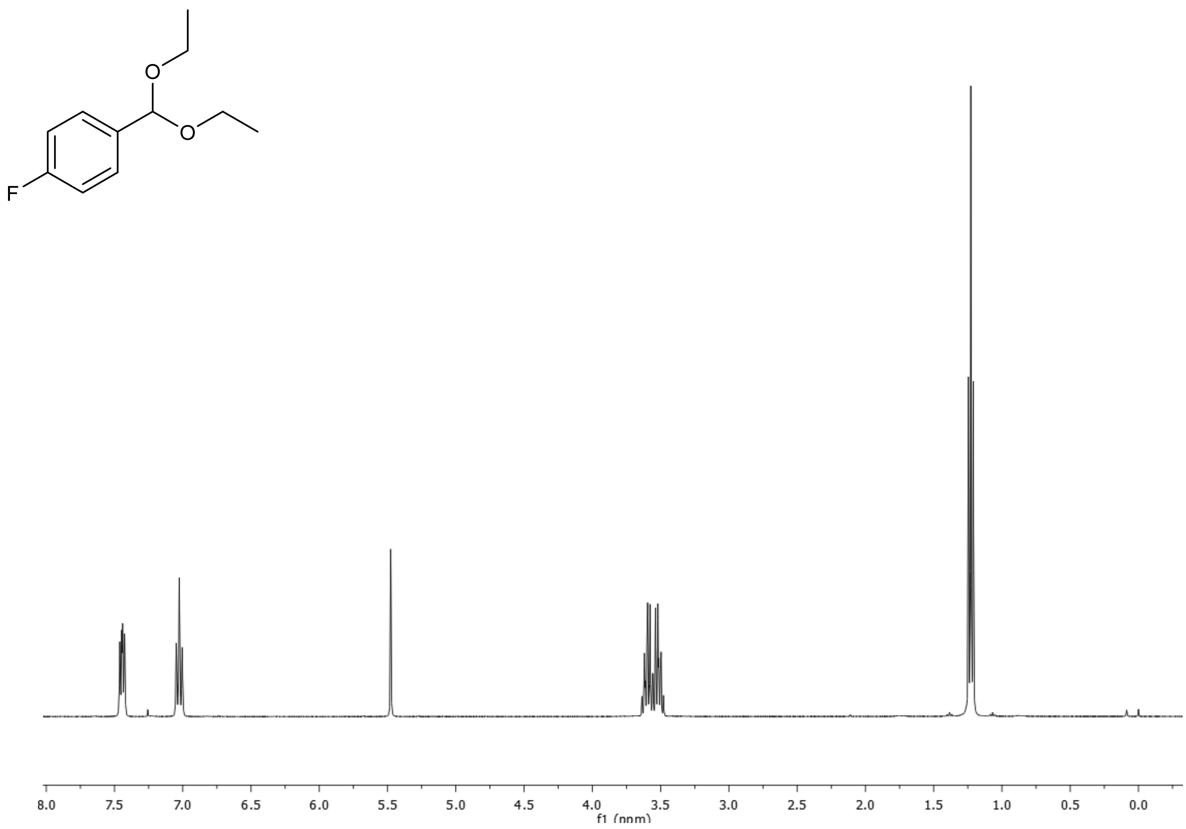

Figure S19. ^{1}H NMR spectrum of product **5** in $CDCl_3$.

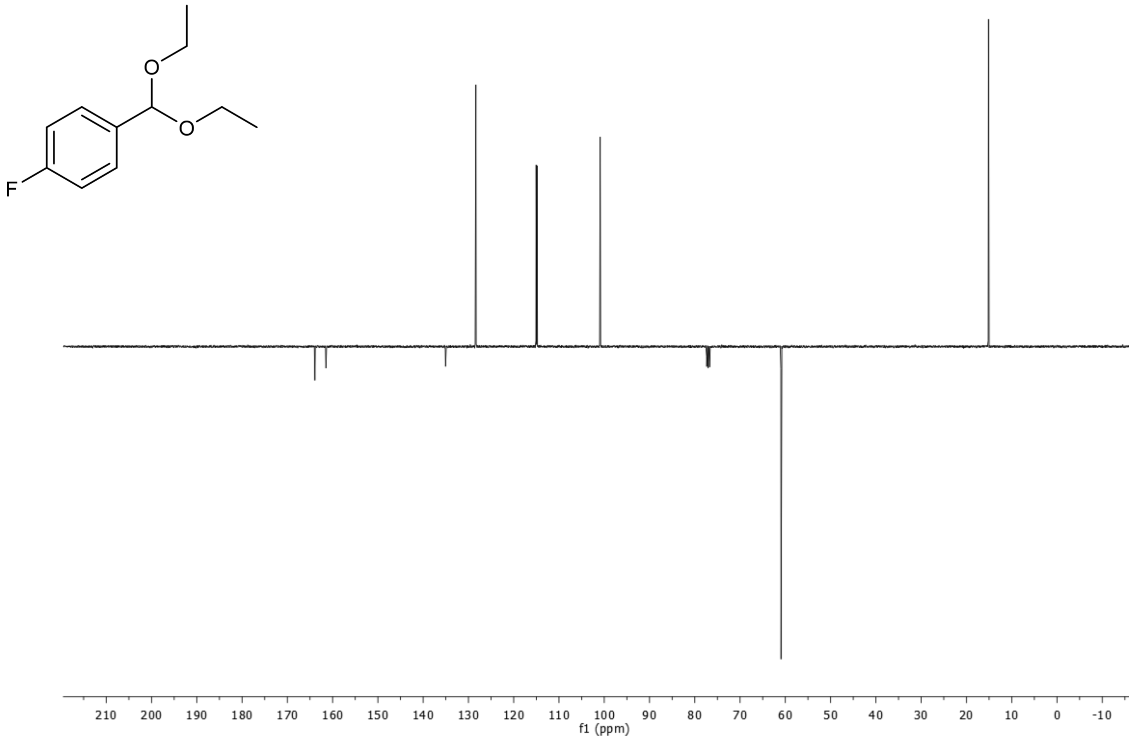

Figure S20. ^{13}C-APT NMR spectrum of product **5** in $CDCl_3$.

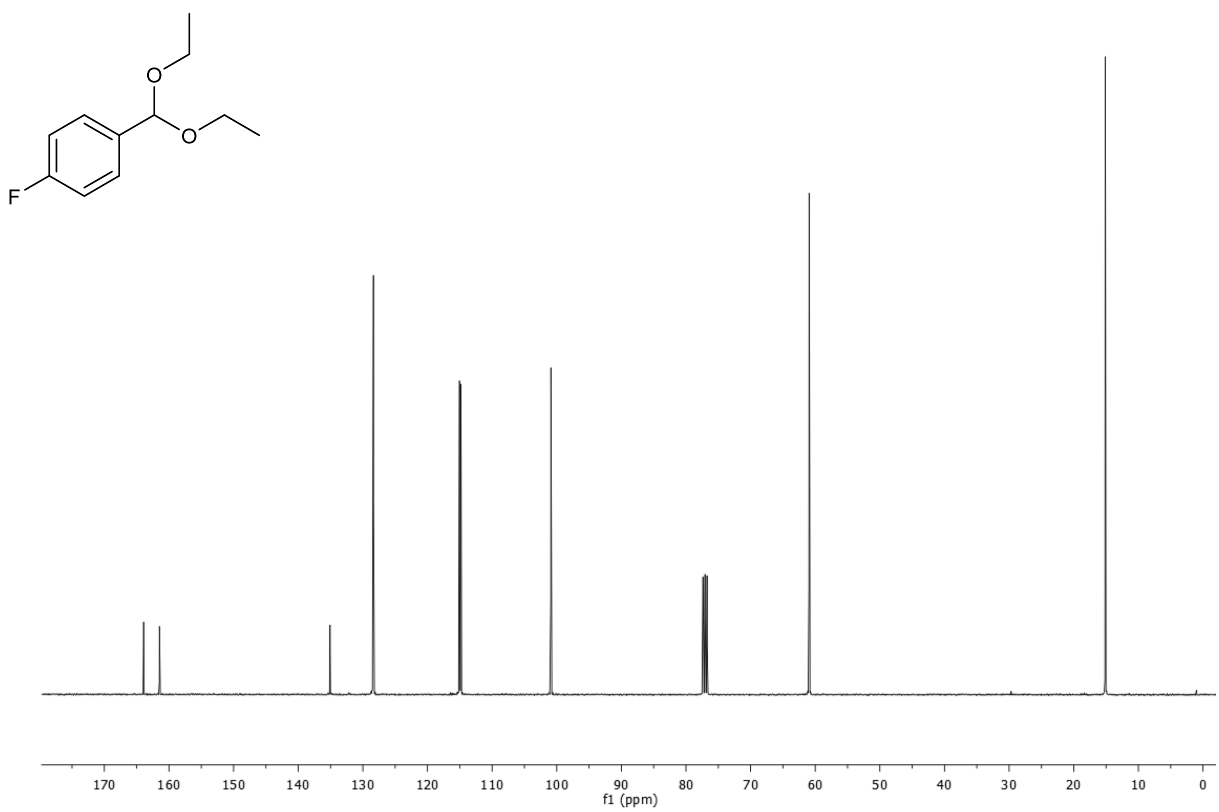

Figure S21. ^{13}C NMR spectrum of product **5** in $CDCl_3$.

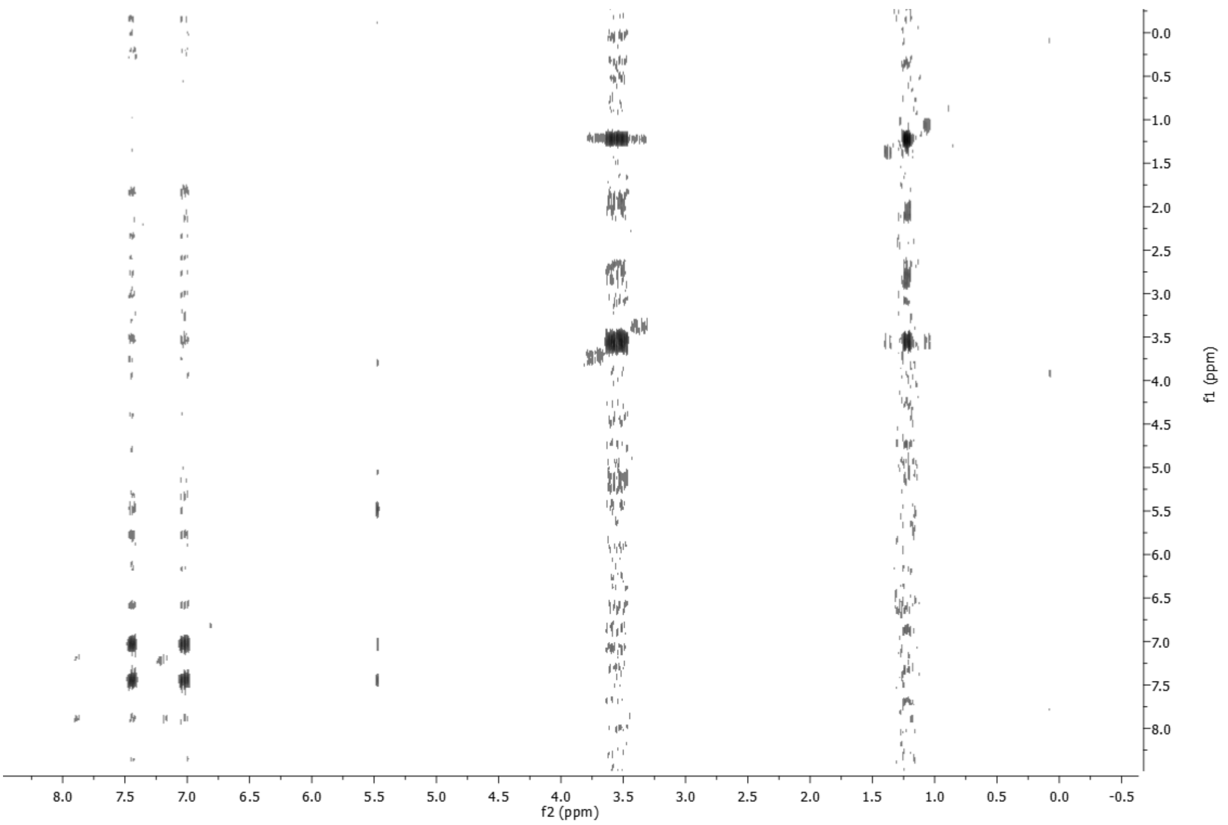

Figure S22. COSY NMR spectrum of product **5** in $CDCl_3$.

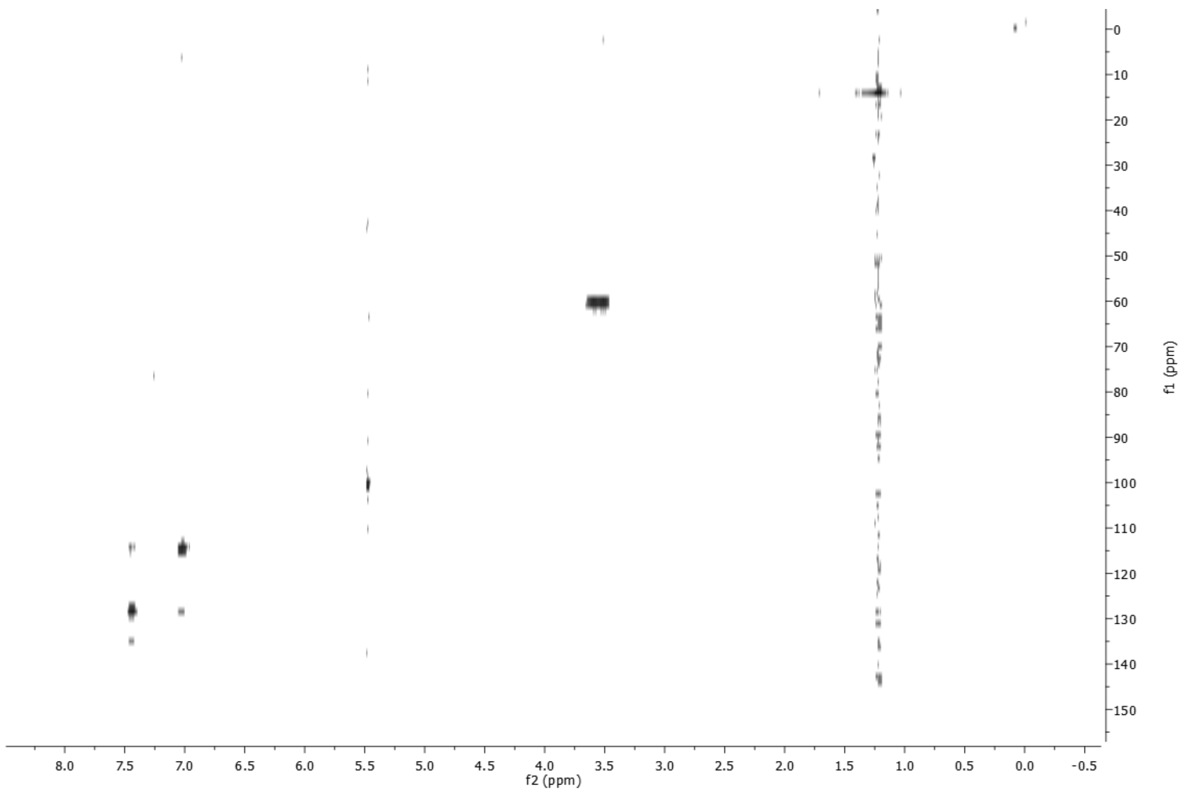

Figure S23. HSQC NMR spectrum of product **5** in $CDCl_3$.

References

1. Wilk, M.; Trzepizur, D.; Koszelewski, D.; Brodzka, A.; Ostaszewski, R. Synthesis of (E)-α,β-unsaturated carboxylic esters derivatives from cyanoacetic acid via promiscuous enzyme-promoted cascade esterification/Knoevenagel reaction. *Bioorg. Chem.* **2019**, doi:10.1016/j.bioorg.2019.02.041.
2. Emsermann, J.; Opatz, T. Photochemical Approaches to the Bilobalide Core. *European J. Org. Chem.* **2017**, doi:10.1002/ejoc.201700461.
3. Xavier, T.; Rayapin, C.; Le Gall, E.; Presset, M. Multicomponent Aromatic and Benzylic Mannich Reactions through C−H Bond Activation. *Chem. - A Eur. J.* **2019**, doi:10.1002/chem.201903414.
4. Spiliopoulou, N.; Nikitas, N.F.; Kokotos, C.G. Photochemical synthesis of acetals utilizing Schreiner's thiourea as the catalyst. *Green Chem.* **2020**, doi:10.1039/d0gc01135e.
5. Baxter, M.; Bolshan, Y. A General Access to Propargylic Ethers through Brønsted Acid Catalyzed Alkynylation of Acetals and Ketals with Trifluoroborates. *Chem. - A Eur. J.* **2015**, doi:10.1002/chem.201502797.
6. Lan, J.; Jiang, G.; Yang, J.; Zhu, H.; Le, Z.; Xie, Z. α-Chymotrypsin-Induced Acetalization of Aldehydes and Ketones with Alcohols. *Synth.* **2020**, doi:10.1055/s-0039-1690883.
7. Subaramanian, M.; Landge, V.G.; Mondal, A.; Gupta, V.; Balaraman, E. Nickel-Catalyzed Chemoselective Acetalization of Aldehydes With Alcohols under Neutral Conditions. *Chem. - An Asian J.* **2019**, doi:10.1002/asia.201900908.
8. Ugarte, R.A.; Hudnall, T.W. Antimony(v) catalyzed acetalisation of aldehydes: An efficient, solvent-free, and recyclable process. *Green Chem.* **2017**, doi:10.1039/c6gc03629e.
9. Maegawa, T.; Otake, K.; Goto, A.; Fujioka, H. Direct conversion of acetals to esters with high regioselectivity via O,P-acetals. *Org. Biomol. Chem.* **2011**, doi:10.1039/c1ob05687e.
10. Silverman, R.B.; Ding, C.Z.; Silverman, R.B.; Ding, C.Z. Chemical Model for a Mechanism of Inactivation of Monoamine Oxidase by Heterocyclic Compounds. Electronic Effects on Acetal Hydrolysis. *J. Am. Chem. Soc.* **1993**, doi:10.1021/ja00064a020.
11. Mensah, E.A.; Green, S.D.; West, J.; Kindoll, T.; Lazaro-Martinez, B. Formation of Acetals and Ketals from Carbonyl Compounds: A New and Highly Efficient Method Inspired by Cationic Palladium. *Synlett* **2019**, doi:10.1055/s-0039-1690497.
12. Du, Y.; Tian, F. Brønsted acidic ionic liquids as efficient and recyclable catalysts for protection of carbonyls to acetals and ketals under mild conditions. *Synth. Commun.* **2005**, doi:10.1080/00397910500214409.
